# Risk of chemotherapy-induced febrile neutropenia in patients with metastatic cancer not receiving granulocyte colony-stimulating factor prophylaxis in US clinical practice

**DOI:** 10.1007/s00520-020-05715-3

**Published:** 2020-09-03

**Authors:** Ahuva Averin, Amanda Silvia, Lois Lamerato, Kathryn Richert-Boe, Manpreet Kaur, Devi Sundaresan, Neel Shah, Mark Hatfield, Tatiana Lawrence, Gary H. Lyman, Derek Weycker

**Affiliations:** 1 Policy Analysis Inc. (PAI), Four Davis Court, Brookline, MA 02445 USA; 2 Henry Ford Health System, Detroit, MI USA; 3 Kaiser Permanente Northwest, Portland, OR USA; 4 Reliant Medical Group, Worcester, MA USA; 5 Amgen Inc., Thousand Oaks, CA USA; 6 Fred Hutchinson Cancer Research Center, Seattle, WA USA

**Keywords:** Granulocyte colony-stimulating factor, Febrile neutropenia, Breast cancer, Colorectal cancer, Lung cancer, Non-Hodgkin lymphoma

## Abstract

**Objectives:**

To evaluate the use of granulocyte colony-stimulating factor (G-CSF) prophylaxis in US patients with selected metastatic cancers and chemotherapy-induced febrile neutropenia (FN) incidence and associated outcomes among the subgroup who did not receive prophylaxis.

**Methods:**

This retrospective cohort study was conducted at four US health systems and included adults with metastatic cancer (breast, colorectal, lung, non-Hodgkin lymphoma [NHL]) who received myelosuppressive chemotherapy (2009–2017). Patients were stratified by FN risk level based on risk factors and chemotherapy (low/unclassified risk, intermediate risk without any risk factors, intermediate risk with ≥ 1 risk factor [IR + 1], high risk [HR]). G-CSF use was evaluated among all patients stratified by FN risk, and FN/FN-related outcomes were evaluated among patients who did not receive first-cycle G-CSF prophylaxis.

**Results:**

Among 1457 metastatic cancer patients, 20.5% and 28.1% were classified as HR and IR + 1, respectively. First-cycle G-CSF prophylaxis use was 48.5% among HR patients and 13.9% among IR + 1 patients. In the subgroup not receiving first-cycle G-CSF prophylaxis, FN incidence in cycle 1 was 7.8% for HR patients and 4.8% for IR + 1 patients; during the course, corresponding values were 16.9% and 15.9%. Most (> 90%) FN episodes required hospitalization, and mortality risk ranged from 7.1 to 26.9% across subgroups.

**Conclusion:**

In this retrospective study, the majority of metastatic cancer chemotherapy patients for whom G-CSF prophylaxis is recommended did not receive it; FN incidence in this subgroup was notably high. Patients with elevated FN risk should be carefully identified and managed to ensure appropriate use of supportive care.

**Electronic supplementary material:**

The online version of this article (10.1007/s00520-020-05715-3) contains supplementary material, which is available to authorized users.

## Introduction

A common challenge in the treatment of nonmyeloid neoplastic disease is the development of chemotherapy-induced neutropenia [1–5], a condition in which the absolute neutrophil count (ANC) drops below normal (< 0.5 × 10^9^/L or < 1.0 × 10^9^/L with a predicted decrease to <0.5 × 10^9^/L) after myelosuppressive chemotherapy [4–7]. Neutropenia is a potentially serious adverse effect that increases the risk of infection and, if untreated, can progress to febrile neutropenia (FN; fever of ≥ 38.3 °C [101 °F]) [6, 7]. FN can lead to chemotherapy dose schedule alterations, increased risk of hospitalization, increased healthcare costs, worse clinical outcomes, and life-threatening complications [1–3, 8, 9].

The risk of developing FN depends on the myelotoxicity of chemotherapy regimens as well as patient and disease characteristics [6, 10]. Therefore, according to National Comprehensive Cancer Network (NCCN) guidelines, prophylactic granulocyte colony-stimulating factor (G-CSF) is recommended for patients undergoing myelosuppressive chemotherapy with a high risk for FN (> 20%) and should be considered in patients with an intermediate risk for FN (10–20%; with ≥ 1 FN risk factor) to reduce the incidence of FN and infection-related complications [6, 11]. G-CSFs increase the production (i.e., differentiation and proliferation) and activity of neutrophils, which improve immune defense against infection and reduce the risk of FN [12–14]. Despite available evidence that prophylactic G-CSF is associated with a lower risk of FN, sustained chemotherapy dose intensity, and reduced mortality [15], several studies have reported that many patients for whom prophylaxis is recommended do not receive it in US clinical practice [15–18].

Failure to administer G-CSF prophylaxis could be especially detrimental in patients with metastatic cancer, who are often older and have more complex comorbidity profiles (vs. non-metastatic patients) and thus for whom the risk of FN may be elevated and the consequences of FN may be more severe [19–21]. The use of intense myelosuppressive chemotherapy with curative intent has become increasingly common in patients with metastatic solid tumors and advanced NHL. Accordingly, updated evidence on the use of G-CSF prophylaxis among metastatic cancer patients for whom it is recommended, and the implications among such patients not receiving prophylaxis, are needed. Therefore, this study was undertaken to benchmark the use of G-CSF prophylaxis and the risk of chemotherapy-induced FN in the absence of G-CSF prophylaxis among patients with metastatic breast cancer, colorectal cancer, lung cancer, and non-Hodgkin lymphoma (NHL) in US clinical practice.

## Methods

### Study design and data source

This study employed a retrospective observational cohort design and was conducted at four US health systems: Geisinger Health System, Henry Ford Health System, Kaiser Permanente Northwest, and Reliant Medical Group (Supplemental Material). From each health system, requisite data spanning 2009 to 2017 were collected from data stores (i.e., administrative databases, electronic medical record systems, cancer registries), as available, and patient medical charts, as needed, using a standardized case report form (CRF; Supplemental Material).

Data collected via the CRF included disease characteristics (e.g., cancer type, cancer stage, diagnosis date), planned and administered chemotherapy (i.e., dose, route, and dates of administration for oral and injectable drugs), use of supportive care (G-CSFs and antimicrobials), FN risk factors (e.g., demographic characteristics, labs, comorbid and pre-existing conditions, measures of health status, treatment history), and study outcomes (e.g., FN, mortality). A master analytic file including data from all four study sites was created and used for analyses described herein. The study was approved by the institutional review boards of all four participating health systems.

### Source and study populations

The source population included all adults who received one or more courses of myelosuppressive chemotherapy for primary breast cancer, colorectal cancer, lung cancer, or NHL from 2009 to 2017 within the four US health systems. From the source population, patients with evidence of metastatic disease were selected for inclusion in the study population. The presence of metastatic disease was identified based on evidence in cancer registries and/or electronic medical records; for patients without definitive information in these two sources, the presence of metastatic disease was determined/confirmed from patient charts. Patients were excluded from the source/study populations if they had > 1 invasive primary cancer (excluding nonmelanoma skin cancer, the same cancer at multiple sites [e.g., bilateral breast cancer], or an invasive cancer of interest and an in situ cancer) before initiation of the first qualifying chemotherapy course or if they had NHL subtypes other than B cell lymphoma. Patients were also excluded if information on the use of healthcare services during the 6-month period before the first qualifying chemotherapy course was incomplete, if their first qualifying chemotherapy course began before the study period, or if they initiated chemotherapy while hospitalized.

### Myelosuppressive chemotherapy

For each patient in the study population, each unique cycle within the first observed full course of myelosuppressive chemotherapy (i.e., the “index course”) was characterized (Supplementary Fig. S1). Chemotherapy regimens were characterized by planned and actual agents, doses, and administration schedule (i.e., weekly [QW], every 2 weeks [Q2W], every 3 weeks [Q3W], every 4 weeks [Q4W]). Chemotherapy regimens were also characterized according to FN risk level (i.e., high, intermediate, low, and unclassified) based on the NCCN guidelines [11] and expert opinion.

### G-CSF prophylaxis

G-CSF prophylaxis was characterized by chemotherapy cycle and was defined as use of filgrastim or pegfilgrastim (including biosimilars) from the first day of chemotherapy administration in a given cycle through the fifth day after completion of chemotherapy administration in that cycle. G-CSF prophylaxis was characterized by agent received, dose, route of administration, timing of administration (pegfilgrastim), and duration of administration (filgrastim). Primary prophylaxis was defined as use beginning in the first cycle, whereas secondary prophylaxis was classified as reactive G-CSF use (i.e., first use during the second cycle or later).

### Febrile neutropenia

FN episodes were ascertained on a cycle-specific basis beginning 6 days after chemotherapy initiation through the last day of the cycle. FN was defined as having an ANC < 1.0 × 10^9^/L and, within 1 day, evidence of infection (body temperature ≥ 38.3 °C [101 °F], infection diagnosis, administration of antimicrobials); neutropenia, fever, or infection diagnosis in the inpatient setting; or neutropenia, fever, or infection diagnosis and, on the same date, evidence of antimicrobial therapy in the outpatient setting. FN-related outcomes were ascertained among patients requiring inpatient care and included hospital length of stay (LOS) and mortality (which was ascertained during the cycle in which the episode occurred).

### FN risk factors

Risk factors for FN included age ≥ 65 years, history of chemotherapy or radiation therapy, history of neutropenia, cancer metastasis to bone, recent surgery, liver dysfunction (i.e., bilirubin > 2.0 mg/dL), and renal dysfunction (i.e., creatinine clearance < 50 mL/min) [11]. Age was determined at initiation of the index chemotherapy course; history of chemotherapy and radiation therapy, any time prior to the course; history of neutropenia, during the 90-day period before the course; and recent surgery, during the 60-day period before the course. Lab values were based on most proximate measurements during the 180-day period before the chemotherapy course.

### Statistical analyses

Patient FN risk factors and chemotherapy FN risk levels were summarized for all patients in the study population, on an overall basis and by cancer type. Use of G-CSF prophylaxis in cycle 1 and during the chemotherapy course was described for all patients and cancer-specific subgroups, respectively, each of which was further stratified by FN risk level: high risk [HR], intermediate risk plus ≥ 1 risk factor [IR + 1], and all others (intermediate risk with no risk factors, low/unclassified risk). Incidence proportions for FN episodes during cycle 1 and the chemotherapy course were calculated for patients who did not receive G-CSF prophylaxis in cycle 1 (all cancers and cancer-specific subgroups, stratified by FN risk level). Outcomes among patients experiencing FN requiring inpatient care were similarly summarized using percentages and means, as appropriate. All analyses were conducted using SAS 9.4 for Windows.

## Results

### Patients

The source population included 4091 patients with breast cancer (*n* = 2007), colorectal cancer (*n* = 697), lung cancer (*n* = 936), or NHL (*n* = 451). Among these patients, 1457 (35.6%) had metastatic disease and were included in the study population: 380 (26.1%) with breast cancer, 360 (24.7%) with colorectal cancer, 626 (43.0%) with lung cancer, and 91 (6.2%) with NHL. Results for the metastatic subgroup are described herein.

Most patients (92.0%) with metastatic disease had ≥ 1 FN risk factor (Table 1). The most common risk factors were renal dysfunction (all cancers: 56.8%; range: 45.3% [breast cancer] to 65.9% [NHL]) and prior chemotherapy or radiation therapy (all cancers: 42.3%; range: 34.2% [colorectal cancer] to 48.4% [lung cancer]). Approximately one-third of patients were aged ≥ 65 years (all cancers: 29.2%; range: 20.8% [breast cancer] to 40.7% [NHL]). Additional patient characteristics are available in Supplemental Table S1.

**Table 1:** FN risk factors and chemotherapy FN risk level among patients with metastatic cancer

	All cancers (*N* = 1457)	Breast cancer (*n* = 380)	Colorectal cancer (*n* = 360)	Lung cancer (*n* = 626)	NHL (*n* = 91)
FN risk factors, %					
Age ≥ 65 years	29.2	20.8	21.1	37.2	40.7
Prior chemotherapy or radiation therapy	42.3	39.2	34.2	48.4	45.1
Prior neutropenia	2.7	1.8	2.2	2.6	9.9
Bone marrow involvement	22.2	27.1	2.8	30.0	24.2
Recent surgery	29.0	40.0	42.2	16.9	14.3
Liver dysfunction (bilirubin > 2.0 mg/dL)	0.8	0.8	1.4	0.5	0
Renal dysfunction (CrCl < 50 mL/min)	56.8	45.3	62.2	59.3	65.9
≥ 1 of the above	92.0	86.3	92.5	95.0	92.3
Chemotherapy FN risk level, %					
High	20.5	45.8	0	17.4	17.6
Intermediate					
≥ 1 FN risk factor	28.1	10.0	48.9	24.8	44.0
0 FN risk factors	2.7	0.5	5.8	1.6	6.6
Low	25.6	8.9	22.5	41.2	0
Unclassified	23.1	34.7	22.8	15.0	31.9

CrCl, creatinine clearance; FN, febrile neutropenia; NHL, non-Hodgkin lymphoma

Nearly half of all patients with metastatic cancer (48.6%) received chemotherapy regimens with a high FN risk level (20.5%) or received regimens with an intermediate FN risk level and had ≥ 1 FN risk factor (28.1%). Breast cancer patients were most likely to receive a chemotherapy regimen with a high FN risk level (45.8%), whereas colorectal patients received only regimens with an intermediate, low, or unclassified FN risk level. Information on commonly administered chemotherapy regimens by cancer type is available in Supplemental Table S2. The frequency of planned chemotherapy regimens and associated FN risk level for patients with breast cancer, colorectal cancer, lung cancer, and NHL are shown in Supplemental Tables S3, S4, S5, and S6, respectively.

### Use of G-CSF prophylaxis

**Fig. 1:**
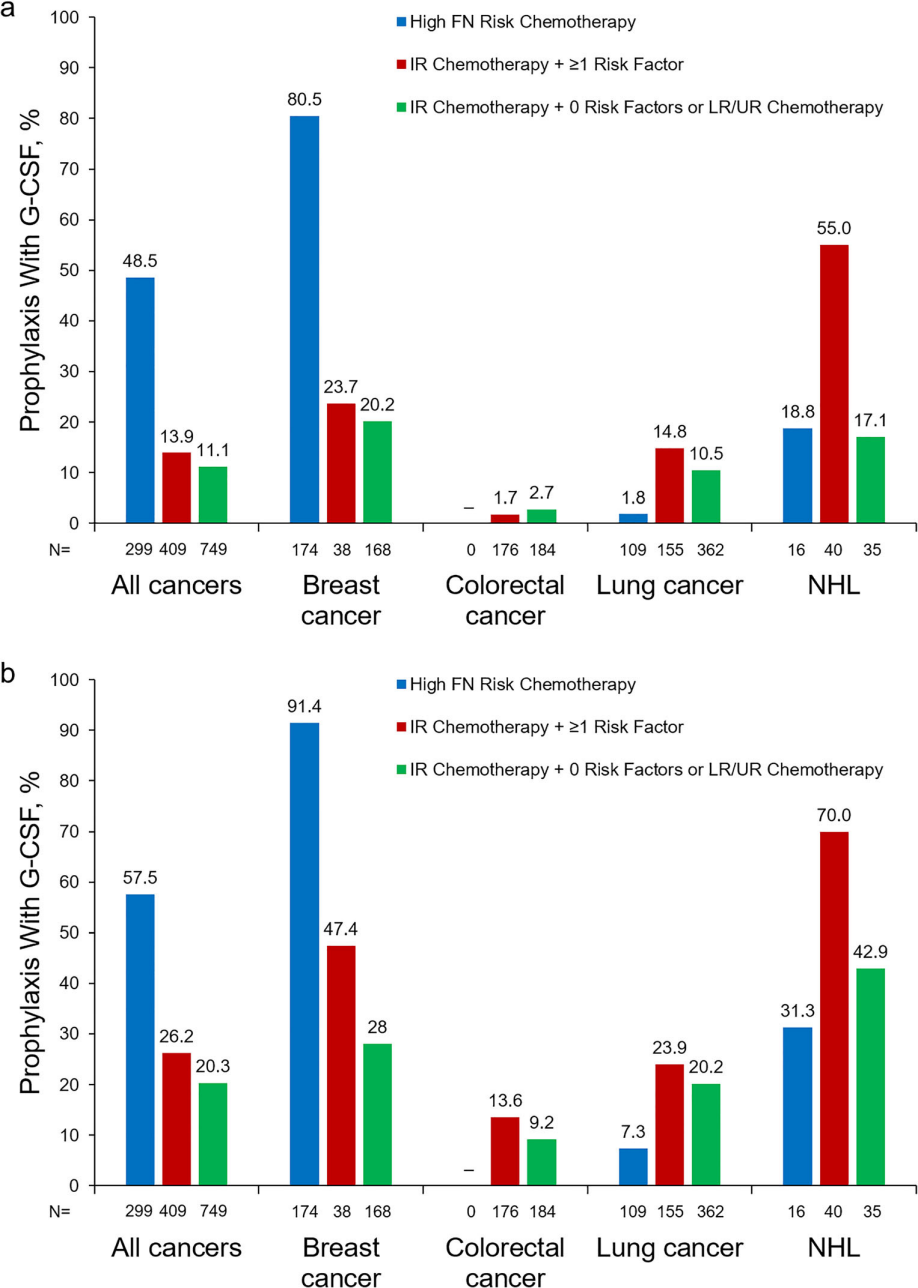
Prophylaxis with G-CSF in all patients with metastatic cancer and patients with metastatic breast cancer, colorectal cancer, lung cancer, and NHL in cycle 1 (**a**) and during the treatment course^a^ (**b**). FN, febrile neutropenia; G-CSF, granulocyte colony-stimulating factor; IR, intermediate FN risk level; LR, low FN risk level; NHL, non-Hodgkin lymphoma; UR, unclassified FN risk level. ^a^Receipt in ≥ 1 cycle during the treatment course

Across all risk categories, 19.6% of patients with metastatic disease were administered prophylactic G-CSF in cycle 1, including 48.5% of HR patients, 13.9% of IR + 1 patients, and 11.1% of all other patients (Fig. 1a). Prophylaxis with G-CSF in cycle 1 ranged from 1.8% (lung cancer) to 80.5% (breast cancer) among HR patients, 14.8% (lung cancer) to 55.0% (NHL) among IR + 1 patients, and 2.7% (colorectal cancer) to 20.2% (breast cancer) among all others. During the chemotherapy course, 57.5% of HR patients, 26.2% of IR + 1 patients, and 20.3% of all other patients received G-CSF prophylaxis in ≥ 1 cycle (Fig. 1b). Course-level use of prophylaxis by cancer type was similar to prophylaxis use in cycle 1. Detailed information on prophylaxis with G-CSF is available in Supplemental Table S7.

### Patients with no G-CSF prophylaxis: FN incidence and outcomes

**Fig. 2:**
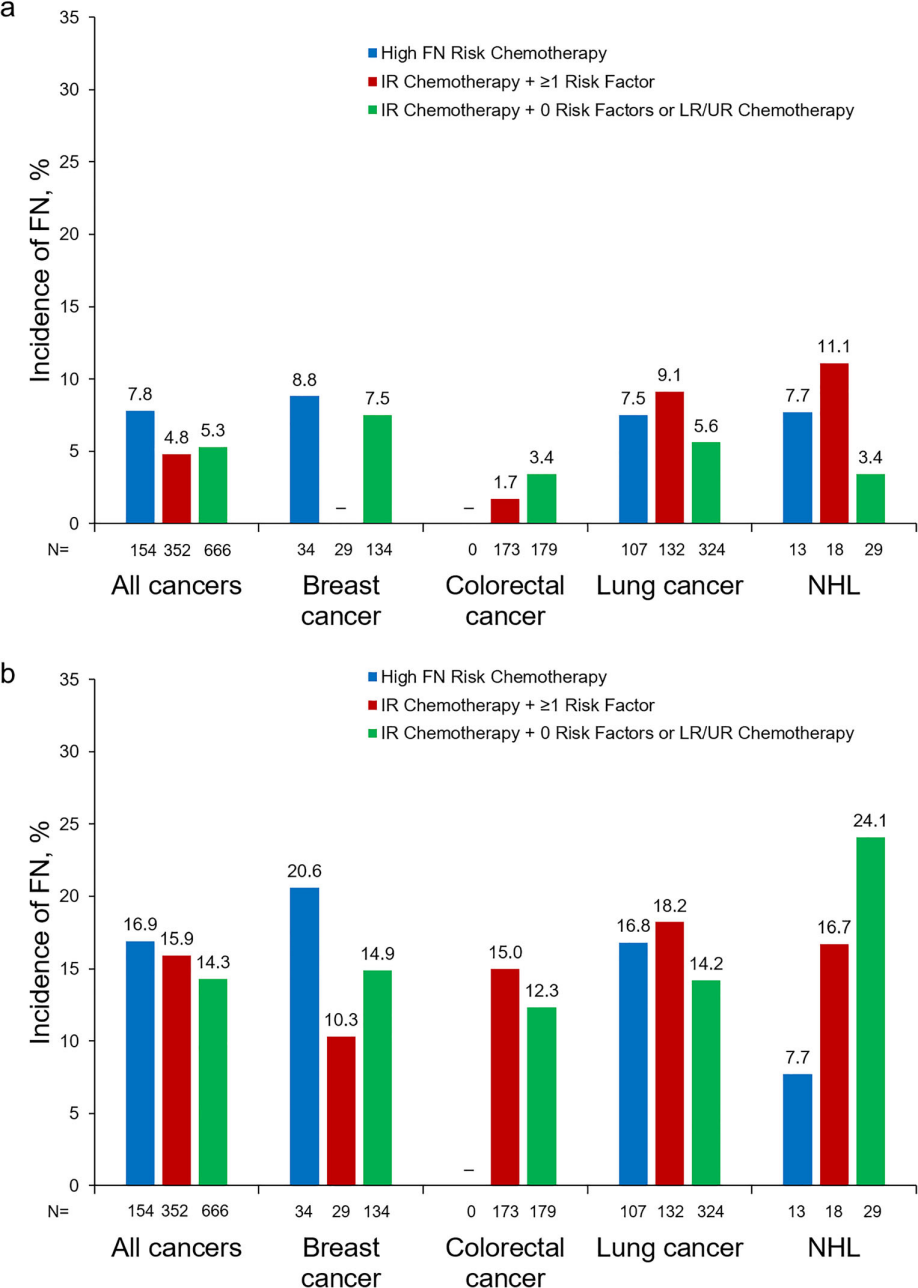
Incidence of FN in patients who did not receive primary prophylactic G-CSF in cycle 1 (**a**) and during the treatment course (**b**) presented by metastatic breast cancer, colorectal cancer, lung cancer, and NHL. FN, febrile neutropenia; IR, intermediate FN risk level; LR, low FN risk level; NHL, non-Hodgkin lymphoma; UR, unclassified FN risk level

Among HR patients who did not receive G-CSF prophylaxis in cycle 1 (*n* = 154/299; 51.5%), FN incidence was 7.8% (range: 7.5% [lung cancer] to 8.8% [breast cancer]) during cycle 1 and 16.9% (range: 7.7% [NHL] to 20.6% [breast cancer]) during the course (Fig. 2a–b). Among IR + 1 patients who did not receive G-CSF prophylaxis in cycle 1 (*n* = 352/409; 86.1%), incidence of FN was 4.8% (range: 0% [breast cancer] to 11.1% [NHL]) during cycle 1 and 15.9% (range: 10.3% [breast cancer] to 18.2% [lung cancer]) during the course. Among all other patients who did not receive G-CSF prophylaxis in cycle 1 (*n* = 666/749; 88.9%), FN incidence was 5.3% (range: 3.4% [colorectal cancer and NHL, each] to 7.5% [breast cancer]) during cycle 1 and 14.3% (range: 12.3% [colorectal cancer] to 24.1% [NHL]) during the course.

Nearly all FN episodes required hospitalization, ranging from 89.3% among IR + 1 patients to 96.2% among HR patients. Mean (SD) hospital LOS ranged from 5.1 (2.8) to 6.7 (5.5) days across subgroups defined on FN risk level, and 7.1 to 26.9% of patients who were hospitalized for FN died during the cycle (Supplemental Table S8).

## Discussion

Therapeutic advances in cancer care during the past several decades have dramatically changed treatment patterns among cancer patients, especially among those with metastatic disease. Palliative care, with correspondingly low rates of survival, has been increasingly replaced with curative treatment, often consisting of multiple lines of therapy [22–24]. Although survival rates have improved, the use of more aggressive myelosuppressive regimens carries considerable risks, including chemotherapy-induced FN, highlighting the increasing importance of G-CSF prophylaxis among patients in this population for whom its use is recommended.

In this retrospective analysis of patients receiving myelosuppressive chemotherapy for metastatic cancer at four US health systems, use of G-CSF prophylaxis varied considerably based on chemotherapy regimen FN risk and presence of patient risk factors for FN, ranging from 13.8% to 48.5% in cycle 1 and 26.2% to 57.5% during the course for IR + 1 and HR patients, respectively. Use of G-CSF prophylaxis varied by cancer type and was greatest among patients with breast cancer and NHL who, consistent with standard of care [11, 25, 26], more commonly received chemotherapy regimens with intermediate or high FN risk (breast cancer: 56.3%; NHL: 68.1%). Previously published studies have also reported low or inconsistent use of G-CSFs for patients with and without metastatic disease, even among those for whom it is recommended [27–29]. For example, a Cancer Care Outcome Research and Surveillance Consortium (CanCORS) study of patients who received chemotherapy for metastatic and non-metastatic lung or colorectal cancers found that CSF use during the course was low regardless of FN regimen risk (10.1%, low; 17.9%, intermediate; 17.2%, high) [27]. In addition, a more recent study found that CSF prophylaxis was administered to only 16.7%, 21.9%, and 9.5% of patients with metastatic breast, lung, and colorectal cancers, respectively, even though regimens with an intermediate or high FN risk level were relatively common among the study population [29]. These findings are also consistent with other published studies [16, 17].

The present study is, to the best of our knowledge, the first to report incidence of FN and FN-related outcomes among patients with metastatic disease stratified by risk level. Our findings suggest that among metastatic cancer patients for whom primary prophylaxis is recommended but not received, FN risk is high (course: HR = 16.9%, IR + 1 = 15.9%; cycle 1: HR = 7.8%, IR + 1 = 4.8%) and associated consequences are severe (> 90% of cases required hospitalization). These results are consistent with those from a recently published study of patients with non-metastatic breast, colorectal, lung, or ovarian cancer or NHL receiving chemotherapy regimens with intermediate/high FN risk (2010–2016), which found that FN incidence during cycle 1 among those not receiving primary prophylaxis with CSF ranged from 3.2% to 5.8% and that over 80% of FN episodes resulted in hospitalization [30]. Additionally, in the aforementioned study of patients with metastatic breast, lung, and colorectal cancer, 13.7% to 20.6% of patients experienced FN during the course, and 88.6% to 93.7% of FN episodes required hospitalization [29]. Taken together, the findings of this study and previous research suggest that the risk of FN and consequences thereof are considerable among patients receiving myelosuppressive chemotherapy for metastatic cancers who do not receive G-CSF prophylaxis.

The current study has several limitations. The first is its retrospective design; because histories are left-truncated and because the accuracy of algorithms/variables capturing patient and treatment characteristics is undoubtedly less than perfect, some patients may have been misclassified in terms of their clinical profile. Furthermore, study outcomes (i.e., G-CSF use, FN risk, and FN-related outcomes) were identified based on all relevant information using clinically appropriate algorithms; however, to the extent that such data were missing and/or algorithms were imperfect, patients may be misclassified and study results may therefore be biased. In addition, FN was identified using all relevant information available (e.g., ANC, diagnoses); however, to the extent that data were missing, the incidence of FN may have been underestimated. The impact of this limitation, however, is believed to be negligible given the availability of data from a variety of different sources at each study site. Finally, because the study population was limited to patients with selected metastatic cancers who received chemotherapy at four US health systems, our study population may not reflect the population of patients treated in clinical practice across the USA; additional research using data from other large populations is needed to validate the applicability and accuracy of the characterization of G-CSF use, FN incidence, and FN-related outcomes reported in this study.

## Conclusions

The findings of this study suggest that a large percentage of metastatic cancer patients receiving myelosuppressive chemotherapy who are candidates for prophylactic G-CSF, per NCCN guidelines, do not receive it. Moreover, among the subset of candidates who do not receive G-CSF, FN incidence during the chemotherapy course is high and associated consequences are severe. As the proportion of patients undergoing curative (vs palliative) chemotherapy for metastatic cancer increases, careful consideration should be given to identifying metastatic cancer patients who are at elevated risk of FN, based on their chemotherapy regimen and risk factors, prior to chemotherapy initiation and throughout the chemotherapy course to ensure appropriate use of supportive care.

## Electronic supplementary material

ESM 1 (PDF 1079 kb)

## Data Availability

The data have been provided by participating health systems and are proprietary, and the authors do not have permission to disseminate the data.
