# Supplementary material for: Risk of chemotherapy-induced febrile neutropenia in patients with metastatic cancer not receiving granulocyte colony-stimulating factor prophylaxis in US clinical practice
Source: Support Care Cancer. 2020 Sep 3;29(4):2179–86. doi: 10.1007/s00520-020-05715-3 (PMC7892737; doi:10.1007/s00520-020-05715-3)
Supplement: Supplementary file 1 — (PDF 1079 kb) [file 520_2020_5715_MOESM1_ESM.pdf]

Supplemental Material

Risk of chemotherapy-induced febrile neutropenia in patients with metastatic cancer not receiving granulocyte colony-stimulating factor prophylaxis in US clinical practice

Authors: Ahuva Hanau,[1] Amanda Silvia,[1] Lois Lamerato,[2] Kathryn Richert-Boe,[3] Manpreet Kaur,[2] Devi Sundaresan,[4] Neel Shah,[5] Mark Hatfield,[5] Tatiana Lawrence,[5] Gary H. Lyman,[6] Derek Weycker[1]

Affiliations: [1]Policy Analysis Inc. (PAI), Brookline, MA, USA; [2]Henry Ford Health System, Detroit, MI, USA; [3]Kaiser Permanente Northwest, Portland, OR, USA; [4]Reliant Medical Group, Worcester, MA, USA; [5]Amgen Inc., Thousand Oaks, CA, USA; [6]Fred Hutchinson Cancer Research Center, Seattle, WA, USA

Corresponding Author:

Derek Weycker, Ph.D.
Policy Analysis Inc. (PAI)
Four Davis Court
Brookline, MA 02445
617-232-4400
dweycker@pai2.com

Case Report Forms and Instructions for Completing Case Report Forms

INSTRUCTIONS FOR COMPLETING CASE REPORT FORMS (CRFs)

1. Print using BLACK or BLUE ballpoint pen only.

2. Print all written entries legibly in BLOCK LETTERS, and use concise language.

3. Use an "X" to mark appropriate boxes, or completely fill in box.

4. Do not write outside the borders of a data entry box.

5. To correct an entry, please use Wite-Out®, <u>OR</u> fill out a new page and replace the old page with the new page. Do NOT draw a line through the data and re-enter a value.

6. Complete only the data requested on each CRF page. Comments and/or additional information should be written only in the space provided for free text or in the area provided for abstractors' comments on the last page of Form A.

7. When completing doses and lab values, include decimal point (if necessary) in its own box.

8. Dates should be written using the following convention: "MM/DD/YYYY" (e.g., January 14, 2016 should be written as 01/14/2016).
   - If a complete date is unknown, enter "00" for the missing month or day (e.g., 01/00/2016, 00/10/2016).

9. Please make certain that the fields "Date Form Completed", "Reviewer ID", and "Patient ID" are completed as required on the CRF:
   - The first digit of the six-digit Patient ID should be "1" for all patients at GHS, "2" for all patients at HFHS, "3" for all patients at KPNW, and "4" for all patients at RMG.
   - Similarly, the first digit of the two-digit Reviewer ID should be "1" for all abstractors at GHS, "2" for all abstractors at HFHS, "3" for all abstractors at KPNW, and "4" for all abstractors at RMG.

10. There are 4 forms (Forms A-D) that could potentially be filled out for each patient.

    Form A:

    This form must be filled out for every patient who qualifies for inclusion in the study population.

    Form B:

    This form must be filled out ONLY if the patient has evidence of a low absolute neutrophil count (ANC) and an elevated body temperature, infection diagnosis, administration of IV AMBs, and/or administration of oral AMBs <u>AND/OR</u> a diagnosis of neutropenia, fever, and/or infection during the INDEX COURSE.

    Form C:

    This form must be filled out ONLY if the patient received colony-stimulating factors (CSFs) at ANY time during the INDEX COURSE.

    Form D:

    This form must be filled out ONLY if the patient received antimicrobials (AMBs) as prophylaxis at ANY time during the INDEX COURSE.

11. For every completed form, all pages of the form must be scanned even if some pages were left blank (e.g., questions about planned and administered chemotherapy may not require all pages to be filled out).

12. If completing forms in batches, each set of forms (e.g., all completed Form As) should be scanned in, saved together, and titled with the date scanned and form name.

SCREENER QUESTIONS
FOR SOURCE POPULATION
(GHS, HFHS, KPNW)

Each patient must satisfy the following criteria—based on data available in machine-searchable electronic medical records—before being considered for inclusion in the study population and proceeding to the next stage of data collection.

1. Did the patient receive ≥1 myelosuppressive chemotherapy agent from January 1, 2015 through June 30, 2017 ("STUDY PERIOD")? The date that the patient first received myelosuppressive chemotherapy during the STUDY PERIOD is designated as the TREATMENT INITIATION DATE.

   If yes (i.e., received chemotherapy), continue to Screening Question #2. If no, stop.

2. For patients with a TREATMENT INITIATION DATE between January 1, 2015 and February 28, 2015, did the patient receive ≥1 myelosuppressive chemotherapy agent during the 60-day period prior to the TREATMENT INITIATION DATE?

   If no (i.e., no prior chemotherapy), continue to Screening Question #3. If yes, stop.

3. Are there ≥6 months of observation recorded in the patient's file before the TREATMENT INITIATION DATE?

   If yes (i.e., evidence of ≥6 months of observation), continue to Screening Question #4. If no, stop.

4. Was there evidence (based on ICD-O or ICD-9 codes) that the patient had invasive breast cancer (female), invasive colorectal cancer, invasive lung cancer, or non-Hodgkin's lymphoma (NHL)?

   If yes (i.e., evidence of a cancer of interest), continue to Screening Question #5. If no, stop.

5. Was the patient in hospital on the TREATMENT INITIATION DATE?

   If no (i.e., not in hospital), continue to Screening Question #6. If yes, stop.

6. Was the patient 18 years of age or older on the TREATMENT INITIATION DATE?

   If yes (i.e., age ≥18 years), continue to Screening Question #7. If no, stop.

7. Was the patient enrolled in a clinical trial for any reason on the TREATMENT INITIATION DATE?

   If no (i.e., not in clinical trial), continue to Screening Question #8. If yes, stop.

8. Did the patient initiate radiation therapy during the 35-day period on or after the TREATMENT INITIATION DATE?

   If no (i.e., did not initiate radiation therapy during the specified time), complete Form A. If yes, stop.

SCREENER QUESTIONS
FOR SOURCE POPULATION
(RMG ONLY)

Each patient must satisfy the following criteria—based on data available in machine-searchable electronic medical records—before being considered for inclusion in the study population and proceeding to the patient's medical chart.

1. Did the patient have ≥1 oncology department encounter from January 1, 2015 through June 30, 2017 ("STUDY PERIOD")?

   If yes (i.e., had an oncology encounter), continue to Screening Question #2. If no, stop.

2. Did the patient have ≥1 encounter diagnosis of any cancer of interest (ICD-9 153.x, 154.x, 162.x, 174.x, 200.x, 202.x) from January 1, 2015 through June 30, 2017?

   If yes (i.e., has a cancer diagnosis), continue to Screening Question #3. If no, stop.

3. Was the patient 18 years of age or older when first receiving myelosuppressive chemotherapy ("TREATMENT INITIATION DATE")?

   If yes (i.e., age ≥18 years), continue to Screening Question #4. If no, stop.

4. Did the patient have at least one encounter with Internal Medicine or the Family Practice Department within six months before the earliest cancer diagnosis encounter during the STUDY PERIOD?

   If yes (i.e., had an encounter with Internal Medicine or the Family Practice Department), proceed to the patient's medical chart and continue to Screening Question #5. If no, stop.

Please proceed to the patient's medical chart. Each patient must satisfy the following criteria before being considered for inclusion in the study population and proceeding to the next stage of data collection.

5. What is the TREATMENT INITIATION DATE during the period of January 1, 2015 through June 30, 2017?

   TREATMENT INITIATION DATE:

Month		Day		Year			

6. For patients with a TREATMENT INITIATION DATE between January 1, 2015 and February 28, 2015, did the patient receive ≥1 myelosuppressive chemotherapy agent during the 60-day period prior to the TREATMENT INITIATION DATE?

   If no (i.e., no prior chemotherapy), continue to Screening Question #7. If yes, stop.

7. Was the patient in hospital on the TREATMENT INITIATION DATE?

   If no (i.e., not in hospital), continue to Screening Question #8. If yes, stop.

8. Was the patient enrolled in a clinical trial for any reason on the TREATMENT INITIATION DATE?

   If no (i.e., not in clinical trial), continue to Screening Question #9. If yes, stop.

9. Did the patient initiate radiation therapy during the 35-day period on or after the TREATMENT INITIATION DATE?

   If no (i.e., did not initiate radiation therapy during the specified time), complete Form A. If yes, stop.

Date Form Completed									Reviewer ID			Patient ID					
Month		Day		Year													

FORM A: CHECKLIST

This page is meant to be used and filled out as the abstractor completes the various forms. This page MUST be filled out and is to be included as the first page of Form A when it is scanned and sent back to PAI.

1. Please complete Form A.

Form A complete? ☐ Yes ⟶ **GO TO Q2**

2. a) Does the patient have evidence of a low absolute neutrophil count (ANC) *AND* an elevated body temperature, infection diagnosis, administration of IV AMBs, and/or administration of oral AMBs on or within one day of the low ANC during the entire chemotherapy course (i.e., from the TREATMENT INITIATION DATE through the last cycle of chemotherapy [up to 18])?
AND/OR
b) Does the patient have any evidence of a diagnosis of neutropenia, fever, and/or infection during the entire chemotherapy course (i.e., from the TREATMENT INITIATION DATE through the last cycle of chemotherapy [up to 18])?

If the answer to both questions is no, do not complete Form B. If the answer to one or both questions is yes, please complete Form B.

☐ No ⟶ **GO TO Q3**

☐ Yes ⟶ **COMPLETE FORM B**

Form B complete? ☐ Yes ⟶ **GO TO Q3**

3. Does patient have evidence of receipt of colony-stimulating factors (CSFs) during the entire chemotherapy course (i.e., from the TREATMENT INITIATION DATE through the last cycle of chemotherapy [up to 18])?

☐ No ⟶ **GO TO Q4**

☐ Yes ⟶ **COMPLETE FORM C**

Form C complete? ☐ Yes ⟶ **GO TO Q4**

4. Does patient have evidence of receipt of antimicrobial (AMB) prophylaxis during the entire chemotherapy course (i.e., from the TREATMENT INITIATION DATE through the last cycle of chemotherapy [up to 18])?

☐ No ⟶ **STOP ABSTRACTION**

☐ Yes ⟶ **COMPLETE FORM D**

Form D complete? ☐ Yes ⟶ **STOP ABSTRACTION**

Date Form Completed							
Month		Day		Year			

Reviewer ID	

Patient ID					

FORM A: SELECTION OF STUDY POPULATION

1. What is the date that the patient first received myelosuppressive chemotherapy ("TREATMENT INITIATION DATE"), during the period of January 1, 2015 through June 30, 2017 ("STUDY PERIOD")? TREATMENT INITIATION DATE marks the first day of the first cycle of the INDEX CHEMOTHERAPY COURSE ("INDEX COURSE").

TREATMENT INITIATION DATE:

Month		Day		Year			

2. According to the Tumor Registry (GHS, HFHS and KPNW) or Medical Chart (RMG), was myelosuppressive chemotherapy administered for a single cancer of interest (invasive breast cancer [female], invasive colorectal cancer, invasive lung cancer, or non-Hodgkin's lymphoma [NHL])? Evidence in the Tumor Registry must be unequivocal, based on diagnosis and other information (e.g., date of diagnosis). If such supporting information is not available or is ambiguous, determination of primary cancer site should be done based on review of Medical Chart.

   NOTE: If a patient has non-melanoma skin cancer and a cancer of interest, OR if the patient has the same cancer at multiple sites (e.g., bilateral breast cancer), OR if the patient has an invasive cancer of interest and an *in situ* cancer, the patient should be included.

   ☐ Yes ⟶ **GO TO Q3**

   ☐ No ⟶ **STOP**

3. Please specify type and date of diagnosis of primary cancer ("DIAGNOSIS DATE"), which should have occurred before the TREATMENT INITIATION DATE.

   ☐ Breast Cancer

   ☐ Colorectal Cancer

   ☐ Lung Cancer

   ☐ NHL

DIAGNOSIS DATE:

Month		Day		Year			

4. Are there ≥6 months of observation recorded in patient's file before the TREATMENT INITIATION DATE?

   ☐ Yes ⟶ **GO TO Q5**

   ☐ No ⟶ **STOP**

5. Does the patient have the NHL subtype B-cell lymphoma (diffuse large B-cell lymphoma and follicular lymphoma)? (For NHL patients only)

   ☐ N/A ⟶ **GO TO Q6**

   ☐ Yes ⟶ **GO TO Q6**

   ☐ No ⟶ **STOP**

FORM A: CHEMOTHERAPY COURSE, CYCLES, AND REGIMEN

Patient ID					

6. Please complete the following information for the PLANNED chemotherapy regimen for the course beginning on the TREATMENT INITIATION DATE of the INDEX COURSE. Please include each agent on a separate line.

	Chemotherapy Agent	Total Dose Planned Per Administration[a]	Dose Unit[b]	Route[c]	Cycle Day(s) of Administration[d]	Cycles Planned[e] (Mark each cycle planned for administration)	Periodicity[f]
a.	______________	☐☐☐☐☐	☐ mg ☐ mg/kg ☐ mg/m² ☐ AUC ☐ Other ______	☐ Oral ☐ IV ☐ SC	______	☐ 1 ☐ 2 ☐ 3 ☐ 4 ☐ 5 ☐ 6 ☐ 7 ☐ 8 ☐ 9 ☐ 10 ☐ 11 ☐ 12 ☐ Other ☐ Unknown	☐ QW ☐ Q2W ☐ Q3W ☐ Q4W ☐ Other ______
b.	______________	☐☐☐☐☐	☐ mg ☐ mg/kg ☐ mg/m² ☐ AUC ☐ Other ______	☐ Oral ☐ IV ☐ SC	______	☐ 1 ☐ 2 ☐ 3 ☐ 4 ☐ 5 ☐ 6 ☐ 7 ☐ 8 ☐ 9 ☐ 10 ☐ 11 ☐ 12 ☐ Other ☐ Unknown	☐ QW ☐ Q2W ☐ Q3W ☐ Q4W ☐ Other ______
c.	______________	☐☐☐☐☐	☐ mg ☐ mg/kg ☐ mg/m² ☐ AUC ☐ Other ______	☐ Oral ☐ IV ☐ SC	______	☐ 1 ☐ 2 ☐ 3 ☐ 4 ☐ 5 ☐ 6 ☐ 7 ☐ 8 ☐ 9 ☐ 10 ☐ 11 ☐ 12 ☐ Other ☐ Unknown	☐ QW ☐ Q2W ☐ Q3W ☐ Q4W ☐ Other ______
d.	______________	☐☐☐☐☐	☐ mg ☐ mg/kg ☐ mg/m² ☐ AUC ☐ Other ______	☐ Oral ☐ IV ☐ SC	______	☐ 1 ☐ 2 ☐ 3 ☐ 4 ☐ 5 ☐ 6 ☐ 7 ☐ 8 ☐ 9 ☐ 10 ☐ 11 ☐ 12 ☐ Other ☐ Unknown	☐ QW ☐ Q2W ☐ Q3W ☐ Q4W ☐ Other ______

[a]Include decimal point (if necessary) in its own box
[b]Dose unit options include: mg, mg/kg, mg/m², AUC, other; if patient's dose unit is not listed above, please write it in. If multiple dose units are available, mg is the preferred unit
[c]Route: IV=intravenous; SC=subcutaneous; if another route was used, please leave boxes blank and provide route in a comment on the Comments page
[d]For cycle day(s) of administration, if chemotherapy agent is administered on multiple days, record each day separated by a comma
[e]Check all boxes for all cycles in which agent was planned (e.g., boxes 1-6 should be checked if agent was planned for cycles 1-6). If "other" or "unknown" checkbox is selected for cycles planned, please provide a comment on the Comments page explaining why selected (i.e., additional cycle(s) planned)
[f]Periodicity options: weekly (QW), every two weeks (Q2W), every three weeks (Q3W), every four weeks (Q4W), other; if periodicity is not listed above, please write it in

FORM A: CHEMOTHERAPY COURSE, CYCLES, AND REGIMEN (CONT.)

Patient ID					

6. ***(Continued)*** Please complete the following information for the PLANNED chemotherapy regimen for the course beginning on the TREATMENT INITIATION DATE of the INDEX COURSE. Please include each agent on a separate line.

	Chemotherapy Agent	Total Dose Planned Per Administration[a]	Dose Unit[b]	Route[c]	Cycle Day(s) of Administration[d]	Cycles Planned[e] (Mark each cycle planned for administration)	Periodicity[f]
e.	______	☐☐☐☐☐	☐ mg ☐ mg/kg ☐ mg/m² ☐ AUC ☐ Other ______	☐ Oral ☐ IV ☐ SC	______	☐ 1 ☐ 2 ☐ 3 ☐ 4 ☐ 5 ☐ 6 ☐ 7 ☐ 8 ☐ 9 ☐ 10 ☐ 11 ☐ 12 ☐ Other ☐ Unknown	☐ QW ☐ Q2W ☐ Q3W ☐ Q4W ☐ Other ______
f.	______	☐☐☐☐☐	☐ mg ☐ mg/kg ☐ mg/m² ☐ AUC ☐ Other ______	☐ Oral ☐ IV ☐ SC	______	☐ 1 ☐ 2 ☐ 3 ☐ 4 ☐ 5 ☐ 6 ☐ 7 ☐ 8 ☐ 9 ☐ 10 ☐ 11 ☐ 12 ☐ Other ☐ Unknown	☐ QW ☐ Q2W ☐ Q3W ☐ Q4W ☐ Other ______
g.	______	☐☐☐☐☐	☐ mg ☐ mg/kg ☐ mg/m² ☐ AUC ☐ Other ______	☐ Oral ☐ IV ☐ SC	______	☐ 1 ☐ 2 ☐ 3 ☐ 4 ☐ 5 ☐ 6 ☐ 7 ☐ 8 ☐ 9 ☐ 10 ☐ 11 ☐ 12 ☐ Other ☐ Unknown	☐ QW ☐ Q2W ☐ Q3W ☐ Q4W ☐ Other ______
h.	______	☐☐☐☐☐	☐ mg ☐ mg/kg ☐ mg/m² ☐ AUC ☐ Other ______	☐ Oral ☐ IV ☐ SC	______	☐ 1 ☐ 2 ☐ 3 ☐ 4 ☐ 5 ☐ 6 ☐ 7 ☐ 8 ☐ 9 ☐ 10 ☐ 11 ☐ 12 ☐ Other ☐ Unknown	☐ QW ☐ Q2W ☐ Q3W ☐ Q4W ☐ Other ______

[a] Include decimal point (if necessary) in its own box

[b] Dose unit options include: mg, mg/kg, mg/m², AUC, other; if patient's dose unit is not listed above, please write it in. If multiple dose units are available, mg is the preferred unit

[c] Route: IV=intravenous; SC=subcutaneous; if another route was used, please leave boxes blank and provide route in a comment on the Comments page

[d] For cycle day(s) of administration, if chemotherapy agent is administered on multiple days, record each day separated by a comma

[e] Check all boxes for all cycles in which agent was planned (e.g., boxes 1-6 should be checked if agent was planned for cycles 1-6). If "other" or "unknown" checkbox is selected for cycles planned, please provide a comment on the Comments page explaining why selected (i.e., additional cycle(s) planned)

[f] Periodicity options: weekly (QW), every two weeks (Q2W), every three weeks (Q3W), every four weeks (Q4W), other; if periodicity is not listed above, please write it in

FORM A: CHEMOTHERAPY COURSE, CYCLES, AND REGIMEN (CONT.)

Patient ID					

7. Please complete the following information on the ACTUAL ADMINISTRATION of chemotherapy during the patient's INDEX COURSE. For each cycle (up to 18), please include each agent on a separate line. All agents administered within 5 days of cycle day 1 (i.e., within the first 6 days of the cycle) are considered part of the cycle regimen. Please include all available information for each cycle (up to 18), even if there was an unplanned switch in the regimen. See below for two examples (FOLFOX and FLOX).

   NOTE: If a second chemotherapy cycle does not commence prior to day 60 of the first cycle, both the first cycle of chemotherapy and the course of chemotherapy will be considered to have been completed on day 35 following the beginning of the cycle. The second and all subsequent cycles of chemotherapy during the INDEX COURSE will be similarly defined.

FOLFOX:
For this patient who received FOLFOX, 12 cycles will be recorded. (Shaded weeks are the ones that will be recorded, each as a different cycle.) For this regimen, fluorouracil was administered at two different doses during each cycle and so both administrations will be recorded.

Agent	Wk 1	Wk 2	Wk 3	Wk 4	Wk 5	Wk 6	Wk 7	Wk 8
Fluorouracil (dose 1)	X	Rest	X	Rest	X	Rest	X	Rest
Fluorouracil (dose 2)	X	Rest	X	Rest	X	Rest	X	Rest
Leucovorin	X	Rest	X	Rest	X	Rest	X	Rest
Oxaliplatin	X	Rest	X	Rest	X	Rest	X	Rest

Agent	Wk 9	Wk 10	Wk 11	Wk 12	Wk 13	Wk 14	Wk 15	Wk 16
Fluorouracil (dose 1)	X	Rest	X	Rest	X	Rest	X	Rest
Fluorouracil (dose 2)	X	Rest	X	Rest	X	Rest	X	Rest
Leucovorin	X	Rest	X	Rest	X	Rest	X	Rest
Oxaliplatin	X	Rest	X	Rest	X	Rest	X	Rest

Agent	Wk 17	Wk 18	Wk 19	Wk 20	Wk 21	Wk 22	Wk 23	Wk 24
Fluorouracil (dose 1)	X	Rest	X	Rest	X	Rest	X	Rest
Fluorouracil (dose 2)	X	Rest	X	Rest	X	Rest	X	Rest
Leucovorin	X	Rest	X	Rest	X	Rest	X	Rest
Oxaliplatin	X	Rest	X	Rest	X	Rest	X	Rest

FLOX:
For this patient who received FLOX, all 18 cycles of the INDEX COURSE will be recorded. (Shaded weeks are the ones that will be recorded, each as a different cycle.)

Agent	Wk 1	Wk 2	Wk 3	Wk 4	Wk 5	Wk 6	Wk 7	Wk 8
Fluorouracil	X	X	X	X	X	X	Rest	Rest
Leucovorin	X	X	X	X	X	X	Rest	Rest
Oxaliplatin	X		X		X		Rest	Rest

Agent	Wk 9	Wk 10	Wk 11	Wk 12	Wk 13	Wk 14	Wk 15	Wk 16
Fluorouracil	X	X	X	X	X	X	Rest	Rest
Leucovorin	X	X	X	X	X	X	Rest	Rest
Oxaliplatin	X		X		X		Rest	Rest

Agent	Wk 17	Wk 18	Wk 19	Wk 20	Wk 21	Wk 22	Wk 23	Wk 24
Fluorouracil	X	X	X	X	X	X	Rest	Rest
Leucovorin	X	X	X	X	X	X	Rest	Rest
Oxaliplatin	X		X		X		Rest	Rest

FORM A: CHEMOTHERAPY COURSE, CYCLES, AND REGIMEN (CONT.)

Patient ID					

7.

ACTUAL ADMINISTRATION OF CHEMOTHERAPY
CYCLE 1

	Chemotherapy Agent	Total Dose Given Per Administration[a]	Dose Unit[b]	Route[c]	Date Agent Administered[d] (Month / Day / Year)	Duration of Therapy[e] (# Days)
a.			☐ mg ☐ mg/kg ☐ mg/m² ☐ AUC ☐ Other ______	☐ Oral ☐ IV ☐ SC	Month: __ __ Day: __ __ Year: __ __ __ __	__ __
b.			☐ mg ☐ mg/kg ☐ mg/m² ☐ AUC ☐ Other ______	☐ Oral ☐ IV ☐ SC	Month: __ __ Day: __ __ Year: __ __ __ __	__ __
c.			☐ mg ☐ mg/kg ☐ mg/m² ☐ AUC ☐ Other ______	☐ Oral ☐ IV ☐ SC	Month: __ __ Day: __ __ Year: __ __ __ __	__ __
d.			☐ mg ☐ mg/kg ☐ mg/m² ☐ AUC ☐ Other ______	☐ Oral ☐ IV ☐ SC	Month: __ __ Day: __ __ Year: __ __ __ __	__ __
e.			☐ mg ☐ mg/kg ☐ mg/m² ☐ AUC ☐ Other ______	☐ Oral ☐ IV ☐ SC	Month: __ __ Day: __ __ Year: __ __ __ __	__ __

[a]Include decimal point (if necessary) in its own box

[b]Dose unit options include: mg, mg/kg, mg/m², AUC, other; if patient's dose unit is not listed above, please write it in. If multiple dose units are available, mg is the preferred unit

[c]Route: IV=intravenous; SC=subcutaneous; if another route was used, please leave boxes blank and provide route in a comment on the Comments page

[d]If chemotherapy agent is administered over multiple days, record first date of administration

[e]Record duration of therapy (total days) for chemotherapy agent

FORM A: CHEMOTHERAPY COURSE, CYCLES, AND REGIMEN (CONT.)

Patient ID					

7. *(continued)*

ACTUAL ADMINISTRATION OF CHEMOTHERAPY
CYCLE 2

☐ Check this box if chemotherapy agent(s), dose(s), dose unit(s), route(s), and duration of therapy are exactly the same as in prior cycle and all agents in this cycle were administered on same date; then complete DATE AGENT ADMINISTERED to the right. If not, complete all fields below.

DATE AGENT ADMINISTERED:

Month		Day		Year			

	Chemotherapy Agent	Total Dose Given Per Administration[a]	Dose Unit[b]	Route[c]	Date Agent Administered[d] (Month / Day / Year)	Duration of Therapy[e] # Days
a.	______	☐☐☐☐☐	☐ mg ☐ mg/kg ☐ mg/m^2 ☐ AUC ☐ Other ______	☐ Oral ☐ IV ☐ SC	Month ☐☐ Day ☐☐ Year ☐☐☐☐	☐☐
b.	______	☐☐☐☐☐	☐ mg ☐ mg/kg ☐ mg/m^2 ☐ AUC ☐ Other ______	☐ Oral ☐ IV ☐ SC	Month ☐☐ Day ☐☐ Year ☐☐☐☐	☐☐
c.	______	☐☐☐☐☐	☐ mg ☐ mg/kg ☐ mg/m^2 ☐ AUC ☐ Other ______	☐ Oral ☐ IV ☐ SC	Month ☐☐ Day ☐☐ Year ☐☐☐☐	☐☐
d.	______	☐☐☐☐☐	☐ mg ☐ mg/kg ☐ mg/m^2 ☐ AUC ☐ Other ______	☐ Oral ☐ IV ☐ SC	Month ☐☐ Day ☐☐ Year ☐☐☐☐	☐☐
e.	______	☐☐☐☐☐	☐ mg ☐ mg/kg ☐ mg/m^2 ☐ AUC ☐ Other ______	☐ Oral ☐ IV ☐ SC	Month ☐☐ Day ☐☐ Year ☐☐☐☐	☐☐

[a]Include decimal point (if necessary) in its own box
[b]Dose unit options include: mg, mg/kg, mg/m^2, AUC, other; if patient's dose unit is not listed above, please write it in. If multiple dose units are available, mg is the preferred unit
[c]Route: IV=intravenous; SC=subcutaneous; if another route was used, please leave boxes blank and provide route in a comment on the Comments page
[d]If chemotherapy agent is administered over multiple days, record first date of administration
[e]Record duration of therapy (total days) for chemotherapy agent

FORM A: CHEMOTHERAPY COURSE, CYCLES, AND REGIMEN (CONT.)

Patient ID					

7. *(continued)*

ACTUAL ADMINISTRATION OF CHEMOTHERAPY
CYCLE 3

☐ Check this box if chemotherapy agent(s), dose(s), dose unit(s), route(s), and duration of therapy are exactly the same as in prior cycle and all agents in this cycle were administered on same date; then complete DATE AGENT ADMINISTERED to the right. If not, complete all fields below.

DATE AGENT ADMINISTERED:

Month		Day		Year			

	Chemotherapy Agent	Total Dose Given Per Administration[a]	Dose Unit[b]	Route[c]	Date Agent Administered[d] (Month / Day / Year)	Duration of Therapy[e] # Days
a.		☐☐☐☐☐	☐ mg ☐ mg/kg ☐ mg/m² ☐ AUC ☐ Other ______	☐ Oral ☐ IV ☐ SC	Month __ __ Day __ __ Year __ __ __ __	__ __
b.		☐☐☐☐☐	☐ mg ☐ mg/kg ☐ mg/m² ☐ AUC ☐ Other ______	☐ Oral ☐ IV ☐ SC	Month __ __ Day __ __ Year __ __ __ __	__ __
c.		☐☐☐☐☐	☐ mg ☐ mg/kg ☐ mg/m² ☐ AUC ☐ Other ______	☐ Oral ☐ IV ☐ SC	Month __ __ Day __ __ Year __ __ __ __	__ __
d.		☐☐☐☐☐	☐ mg ☐ mg/kg ☐ mg/m² ☐ AUC ☐ Other ______	☐ Oral ☐ IV ☐ SC	Month __ __ Day __ __ Year __ __ __ __	__ __
e.		☐☐☐☐☐	☐ mg ☐ mg/kg ☐ mg/m² ☐ AUC ☐ Other ______	☐ Oral ☐ IV ☐ SC	Month __ __ Day __ __ Year __ __ __ __	__ __

[a]Include decimal point (if necessary) in its own box

[b]Dose unit options include: mg, mg/kg, mg/m², AUC, other; if patient's dose unit is not listed above, please write it in. If multiple dose units are available, mg is the preferred unit

[c]Route: IV=intravenous; SC=subcutaneous; if another route was used, please leave boxes blank and provide route in a comment on the Comments page

[d]If chemotherapy agent is administered over multiple days, record first date of administration

[e]Record duration of therapy (total days) for chemotherapy agent

FORM A: CHEMOTHERAPY COURSE, CYCLES, AND REGIMEN (CONT.)

Patient ID					

7. *(continued)*

ACTUAL ADMINISTRATION OF CHEMOTHERAPY
CYCLE 4

☐ Check this box if chemotherapy agent(s), dose(s), dose unit(s), route(s), and duration of therapy are exactly the same as in prior cycle and all agents in this cycle were administered on same date; then complete DATE AGENT ADMINISTERED to the right. If not, complete all fields below.

DATE AGENT ADMINISTERED:

Month		Day		Year			

	Chemotherapy Agent	Total Dose Given Per Administration[a]	Dose Unit[b]	Route[c]	Date Agent Administered[d] (Month / Day / Year)	Duration of Therapy[e] # Days
a.			☐ mg ☐ mg/kg ☐ mg/m² ☐ AUC ☐ Other ______	☐ Oral ☐ IV ☐ SC		
b.			☐ mg ☐ mg/kg ☐ mg/m² ☐ AUC ☐ Other ______	☐ Oral ☐ IV ☐ SC		
c.			☐ mg ☐ mg/kg ☐ mg/m² ☐ AUC ☐ Other ______	☐ Oral ☐ IV ☐ SC		
d.			☐ mg ☐ mg/kg ☐ mg/m² ☐ AUC ☐ Other ______	☐ Oral ☐ IV ☐ SC		
e.			☐ mg ☐ mg/kg ☐ mg/m² ☐ AUC ☐ Other ______	☐ Oral ☐ IV ☐ SC		

[a]Include decimal point (if necessary) in its own box

[b]Dose unit options include: mg, mg/kg, mg/m², AUC, other; if patient's dose unit is not listed above, please write it in. If multiple dose units are available, mg is the preferred unit

[c]Route: IV=intravenous; SC=subcutaneous; if another route was used, please leave boxes blank and provide route in a comment on the Comments page

[d]If chemotherapy agent is administered over multiple days, record first date of administration

[e]Record duration of therapy (total days) for chemotherapy agent

FORM A: CHEMOTHERAPY COURSE, CYCLES, AND REGIMEN (CONT.)

Patient ID					

7. *(continued)*

ACTUAL ADMINISTRATION OF CHEMOTHERAPY
CYCLE 5

☐ Check this box if chemotherapy agent(s), dose(s), dose unit(s), route(s), and duration of therapy are exactly the same as in prior cycle and all agents in this cycle were administered on same date; then complete DATE AGENT ADMINISTERED to the right. If not, complete all fields below.

DATE AGENT ADMINISTERED:

Month		Day		Year			

	Chemotherapy Agent	Total Dose Given Per Administration[a]	Dose Unit[b]	Route[c]	Date Agent Administered[d] (Month / Day / Year)	Duration of Therapy[e] (# Days)
a.			☐ mg ☐ mg/kg ☐ mg/m² ☐ AUC ☐ Other ______	☐ Oral ☐ IV ☐ SC		
b.			☐ mg ☐ mg/kg ☐ mg/m² ☐ AUC ☐ Other ______	☐ Oral ☐ IV ☐ SC		
c.			☐ mg ☐ mg/kg ☐ mg/m² ☐ AUC ☐ Other ______	☐ Oral ☐ IV ☐ SC		
d.			☐ mg ☐ mg/kg ☐ mg/m² ☐ AUC ☐ Other ______	☐ Oral ☐ IV ☐ SC		
e.			☐ mg ☐ mg/kg ☐ mg/m² ☐ AUC ☐ Other ______	☐ Oral ☐ IV ☐ SC		

[a]Include decimal point (if necessary) in its own box

[b]Dose unit options include: mg, mg/kg, mg/m², AUC, other; if patient's dose unit is not listed above, please write it in. If multiple dose units are available, mg is the preferred unit

[c]Route: IV=intravenous; SC=subcutaneous; if another route was used, please leave boxes blank and provide route in a comment on the Comments page

[d]If chemotherapy agent is administered over multiple days, record first date of administration

[e]Record duration of therapy (total days) for chemotherapy agent

FORM A: CHEMOTHERAPY COURSE, CYCLES, AND REGIMEN (CONT.)

Patient ID					

7. *(continued)*

ACTUAL ADMINISTRATION OF CHEMOTHERAPY
CYCLE 6

☐ Check this box if chemotherapy agent(s), dose(s), dose unit(s), route(s), and duration of therapy are exactly the same as in prior cycle and all agents in this cycle were administered on same date; then complete DATE AGENT ADMINISTERED to the right. If not, complete all fields below.

DATE AGENT ADMINISTERED:

Month		Day		Year			

	Chemotherapy Agent	Total Dose Given Per Administration[a]	Dose Unit[b]	Route[c]	Date Agent Administered[d] (Month / Day / Year)	Duration of Therapy[e] # Days
a.			☐ mg ☐ mg/kg ☐ mg/m² ☐ AUC ☐ Other ______	☐ Oral ☐ IV ☐ SC		
b.			☐ mg ☐ mg/kg ☐ mg/m² ☐ AUC ☐ Other ______	☐ Oral ☐ IV ☐ SC		
c.			☐ mg ☐ mg/kg ☐ mg/m² ☐ AUC ☐ Other ______	☐ Oral ☐ IV ☐ SC		
d.			☐ mg ☐ mg/kg ☐ mg/m² ☐ AUC ☐ Other ______	☐ Oral ☐ IV ☐ SC		
e.			☐ mg ☐ mg/kg ☐ mg/m² ☐ AUC ☐ Other ______	☐ Oral ☐ IV ☐ SC		

[a]Include decimal point (if necessary) in its own box

[b]Dose unit options include: mg, mg/kg, mg/m², AUC, other; if patient's dose unit is not listed above, please write it in. If multiple dose units are available, mg is the preferred unit

[c]Route: IV=intravenous; SC=subcutaneous; if another route was used, please leave boxes blank and provide route in a comment on the Comments page

[d]If chemotherapy agent is administered over multiple days, record first date of administration

[e]Record duration of therapy (total days) for chemotherapy agent

FORM A: CHEMOTHERAPY COURSE, CYCLES, AND REGIMEN (CONT.)

Patient ID					

7. *(continued)*

ACTUAL ADMINISTRATION OF CHEMOTHERAPY
CYCLE 7

☐ Check this box if chemotherapy agent(s), dose(s), dose unit(s), route(s), and duration of therapy are exactly the same as in prior cycle and all agents in this cycle were administered on same date; then complete DATE AGENT ADMINISTERED to the right. If not, complete all fields below.

DATE AGENT ADMINISTERED:

Month		Day		Year			

	Chemotherapy Agent	Total Dose Given Per Administration[a]	Dose Unit[b]	Route[c]	Date Agent Administered[d] (Month / Day / Year)	Duration of Therapy[e] (# Days)
a.			☐ mg ☐ mg/kg ☐ mg/m² ☐ AUC ☐ Other ______	☐ Oral ☐ IV ☐ SC		
b.			☐ mg ☐ mg/kg ☐ mg/m² ☐ AUC ☐ Other ______	☐ Oral ☐ IV ☐ SC		
c.			☐ mg ☐ mg/kg ☐ mg/m² ☐ AUC ☐ Other ______	☐ Oral ☐ IV ☐ SC		
d.			☐ mg ☐ mg/kg ☐ mg/m² ☐ AUC ☐ Other ______	☐ Oral ☐ IV ☐ SC		
e.			☐ mg ☐ mg/kg ☐ mg/m² ☐ AUC ☐ Other ______	☐ Oral ☐ IV ☐ SC		

[a]Include decimal point (if necessary) in its own box
[b]Dose unit options include: mg, mg/kg, mg/m², AUC, other; if patient's dose unit is not listed above, please write it in. If multiple dose units are available, mg is the preferred unit
[c]Route: IV=intravenous; SC=subcutaneous; if another route was used, please leave boxes blank and provide route in a comment on the Comments page
[d]If chemotherapy agent is administered over multiple days, record first date of administration
[e]Record duration of therapy (total days) for chemotherapy agent

FORM A: CHEMOTHERAPY COURSE, CYCLES, AND REGIMEN (CONT.)

Patient ID					

7. *(continued)*

ACTUAL ADMINISTRATION OF CHEMOTHERAPY
CYCLE 8

☐ Check this box if chemotherapy agent(s), dose(s), dose unit(s), route(s), and duration of therapy are exactly the same as in prior cycle and all agents in this cycle were administered on same date; then complete DATE AGENT ADMINISTERED to the right. If not, complete all fields below.

DATE AGENT ADMINISTERED:

Month		Day		Year			

	Chemotherapy Agent	Total Dose Given Per Administration[a]	Dose Unit[b]	Route[c]	Date Agent Administered[d] (Month / Day / Year)	Duration of Therapy[e] # Days
a.		☐☐☐☐☐	☐ mg ☐ mg/kg ☐ mg/m² ☐ AUC ☐ Other ______	☐ Oral ☐ IV ☐ SC	Month ☐☐ Day ☐☐ Year ☐☐☐☐	☐☐
b.		☐☐☐☐☐	☐ mg ☐ mg/kg ☐ mg/m² ☐ AUC ☐ Other ______	☐ Oral ☐ IV ☐ SC	Month ☐☐ Day ☐☐ Year ☐☐☐☐	☐☐
c.		☐☐☐☐☐	☐ mg ☐ mg/kg ☐ mg/m² ☐ AUC ☐ Other ______	☐ Oral ☐ IV ☐ SC	Month ☐☐ Day ☐☐ Year ☐☐☐☐	☐☐
d.		☐☐☐☐☐	☐ mg ☐ mg/kg ☐ mg/m² ☐ AUC ☐ Other ______	☐ Oral ☐ IV ☐ SC	Month ☐☐ Day ☐☐ Year ☐☐☐☐	☐☐
e.		☐☐☐☐☐	☐ mg ☐ mg/kg ☐ mg/m² ☐ AUC ☐ Other ______	☐ Oral ☐ IV ☐ SC	Month ☐☐ Day ☐☐ Year ☐☐☐☐	☐☐

[a]Include decimal point (if necessary) in its own box

[b]Dose unit options include: mg, mg/kg, mg/m², AUC, other; if patient's dose unit is not listed above, please write it in. If multiple dose units are available, mg is the preferred unit

[c]Route: IV=intravenous; SC=subcutaneous; if another route was used, please leave boxes blank and provide route in a comment on the Comments page

[d]If chemotherapy agent is administered over multiple days, record first date of administration

[e]Record duration of therapy (total days) for chemotherapy agent

FORM A: CHEMOTHERAPY COURSE, CYCLES, AND REGIMEN (CONT.)

Patient ID					

7. *(continued)*

ACTUAL ADMINISTRATION OF CHEMOTHERAPY
CYCLE 9

☐ Check this box if chemotherapy agent(s), dose(s), dose unit(s), route(s), and duration of therapy are exactly the same as in prior cycle and all agents in this cycle were administered on same date; then complete DATE AGENT ADMINISTERED to the right. If not, complete all fields below.

DATE AGENT ADMINISTERED:

Month		Day		Year			

	Chemotherapy Agent	Total Dose Given Per Administration[a]	Dose Unit[b]	Route[c]	Date Agent Administered[d] (Month / Day / Year)	Duration of Therapy[e] (# Days)
a.			☐ mg ☐ mg/kg ☐ mg/m² ☐ AUC ☐ Other ______	☐ Oral ☐ IV ☐ SC		
b.			☐ mg ☐ mg/kg ☐ mg/m² ☐ AUC ☐ Other ______	☐ Oral ☐ IV ☐ SC		
c.			☐ mg ☐ mg/kg ☐ mg/m² ☐ AUC ☐ Other ______	☐ Oral ☐ IV ☐ SC		
d.			☐ mg ☐ mg/kg ☐ mg/m² ☐ AUC ☐ Other ______	☐ Oral ☐ IV ☐ SC		
e.			☐ mg ☐ mg/kg ☐ mg/m² ☐ AUC ☐ Other ______	☐ Oral ☐ IV ☐ SC		

[a]Include decimal point (if necessary) in its own box
[b]Dose unit options include: mg, mg/kg, mg/m², AUC, other; if patient's dose unit is not listed above, please write it in. If multiple dose units are available, mg is the preferred unit
[c]Route: IV=intravenous; SC=subcutaneous; if another route was used, please leave boxes blank and provide route in a comment on the Comments page
[d]If chemotherapy agent is administered over multiple days, record first date of administration
[e]Record duration of therapy (total days) for chemotherapy agent

FORM A: CHEMOTHERAPY COURSE, CYCLES, AND REGIMEN (CONT.)

Patient ID					

7. *(continued)*

ACTUAL ADMINISTRATION OF CHEMOTHERAPY
CYCLE 10

☐ Check this box if chemotherapy agent(s), dose(s), dose unit(s), route(s), and duration of therapy are exactly the same as in prior cycle and all agents in this cycle were administered on same date; then complete DATE AGENT ADMINISTERED to the right. If not, complete all fields below.

DATE AGENT ADMINISTERED:

Month		Day		Year			

	Chemotherapy Agent	Total Dose Given Per Administration[a]	Dose Unit[b]	Route[c]	Date Agent Administered[d] (Month / Day / Year)	Duration of Therapy[e] # Days
a.			☐ mg ☐ mg/kg ☐ mg/m² ☐ AUC ☐ Other ______	☐ Oral ☐ IV ☐ SC		
b.			☐ mg ☐ mg/kg ☐ mg/m² ☐ AUC ☐ Other ______	☐ Oral ☐ IV ☐ SC		
c.			☐ mg ☐ mg/kg ☐ mg/m² ☐ AUC ☐ Other ______	☐ Oral ☐ IV ☐ SC		
d.			☐ mg ☐ mg/kg ☐ mg/m² ☐ AUC ☐ Other ______	☐ Oral ☐ IV ☐ SC		
e.			☐ mg ☐ mg/kg ☐ mg/m² ☐ AUC ☐ Other ______	☐ Oral ☐ IV ☐ SC		

[a]Include decimal point (if necessary) in its own box
[b]Dose unit options include: mg, mg/kg, mg/m², AUC, other; if patient's dose unit is not listed above, please write it in. If multiple dose units are available, mg is the preferred unit
[c]Route: IV=intravenous; SC=subcutaneous; if another route was used, please leave boxes blank and provide route in a comment on the Comments page
[d]If chemotherapy agent is administered over multiple days, record first date of administration
[e]Record duration of therapy (total days) for chemotherapy agent

FORM A: CHEMOTHERAPY COURSE, CYCLES, AND REGIMEN (CONT.)

Patient ID					

7. *(continued)*

ACTUAL ADMINISTRATION OF CHEMOTHERAPY
CYCLE 11

☐ Check this box if chemotherapy agent(s), dose(s), dose unit(s), route(s), and duration of therapy are exactly the same as in prior cycle and all agents in this cycle were administered on same date; then complete DATE AGENT ADMINISTERED to the right. If not, complete all fields below.

DATE AGENT ADMINISTERED:

Month		Day		Year			

	Chemotherapy Agent	Total Dose Given Per Administration[a]	Dose Unit[b]	Route[c]	Date Agent Administered[d] (Month / Day / Year)	Duration of Therapy[e] # Days
a.			☐ mg ☐ mg/kg ☐ mg/m² ☐ AUC ☐ Other ______	☐ Oral ☐ IV ☐ SC		
b.			☐ mg ☐ mg/kg ☐ mg/m² ☐ AUC ☐ Other ______	☐ Oral ☐ IV ☐ SC		
c.			☐ mg ☐ mg/kg ☐ mg/m² ☐ AUC ☐ Other ______	☐ Oral ☐ IV ☐ SC		
d.			☐ mg ☐ mg/kg ☐ mg/m² ☐ AUC ☐ Other ______	☐ Oral ☐ IV ☐ SC		
e.			☐ mg ☐ mg/kg ☐ mg/m² ☐ AUC ☐ Other ______	☐ Oral ☐ IV ☐ SC		

[a]Include decimal point (if necessary) in its own box

[b]Dose unit options include: mg, mg/kg, mg/m², AUC, other; if patient's dose unit is not listed above, please write it in. If multiple dose units are available, mg is the preferred unit

[c]Route: IV=intravenous; SC=subcutaneous; if another route was used, please leave boxes blank and provide route in a comment on the Comments page

[d]If chemotherapy agent is administered over multiple days, record first date of administration

[e]Record duration of therapy (total days) for chemotherapy agent

FORM A: CHEMOTHERAPY COURSE, CYCLES, AND REGIMEN (CONT.)

Patient ID					

7. *(continued)*

ACTUAL ADMINISTRATION OF CHEMOTHERAPY
CYCLE 12

☐ Check this box if chemotherapy agent(s), dose(s), dose unit(s), route(s), and duration of therapy are exactly the same as in prior cycle and all agents in this cycle were administered on same date; then complete DATE AGENT ADMINISTERED to the right. If not, complete all fields below.

DATE AGENT ADMINISTERED:

Month		Day		Year			

	Chemotherapy Agent	Total Dose Given Per Administration[a]	Dose Unit[b]	Route[c]	Date Agent Administered[d] (Month / Day / Year)	Duration of Therapy[e] # Days
a.			☐ mg ☐ mg/kg ☐ mg/m² ☐ AUC ☐ Other ______	☐ Oral ☐ IV ☐ SC		
b.			☐ mg ☐ mg/kg ☐ mg/m² ☐ AUC ☐ Other ______	☐ Oral ☐ IV ☐ SC		
c.			☐ mg ☐ mg/kg ☐ mg/m² ☐ AUC ☐ Other ______	☐ Oral ☐ IV ☐ SC		
d.			☐ mg ☐ mg/kg ☐ mg/m² ☐ AUC ☐ Other ______	☐ Oral ☐ IV ☐ SC		
e.			☐ mg ☐ mg/kg ☐ mg/m² ☐ AUC ☐ Other ______	☐ Oral ☐ IV ☐ SC		

[a]Include decimal point (if necessary) in its own box
[b]Dose unit options include: mg, mg/kg, mg/m², AUC, other; if patient's dose unit is not listed above, please write it in. If multiple dose units are available, mg is the preferred unit
[c]Route: IV=intravenous; SC=subcutaneous; if another route was used, please leave boxes blank and provide route in a comment on the Comments page
[d]If chemotherapy agent is administered over multiple days, record first date of administration
[e]Record duration of therapy (total days) for chemotherapy agent

FORM A: CHEMOTHERAPY COURSE, CYCLES, AND REGIMEN (CONT.)

Patient ID					

7. *(continued)*

ACTUAL ADMINISTRATION OF CHEMOTHERAPY
CYCLE 13

☐ Check this box if chemotherapy agent(s), dose(s), dose unit(s), route(s), and duration of therapy are exactly the same as in prior cycle and all agents in this cycle were administered on same date; then complete DATE AGENT ADMINISTERED to the right. If not, complete all fields below.

DATE AGENT ADMINISTERED:

Month		Day		Year			

	Chemotherapy Agent	Total Dose Given Per Administration[a]	Dose Unit[b]	Route[c]	Date Agent Administered[d] (Month / Day / Year)	Duration of Therapy[e] # Days
a.			☐ mg ☐ mg/kg ☐ mg/m² ☐ AUC ☐ Other ____	☐ Oral ☐ IV ☐ SC	Month: __ __ Day: __ __ Year: __ __ __ __	__ __
b.			☐ mg ☐ mg/kg ☐ mg/m² ☐ AUC ☐ Other ____	☐ Oral ☐ IV ☐ SC	Month: __ __ Day: __ __ Year: __ __ __ __	__ __
c.			☐ mg ☐ mg/kg ☐ mg/m² ☐ AUC ☐ Other ____	☐ Oral ☐ IV ☐ SC	Month: __ __ Day: __ __ Year: __ __ __ __	__ __
d.			☐ mg ☐ mg/kg ☐ mg/m² ☐ AUC ☐ Other ____	☐ Oral ☐ IV ☐ SC	Month: __ __ Day: __ __ Year: __ __ __ __	__ __
e.			☐ mg ☐ mg/kg ☐ mg/m² ☐ AUC ☐ Other ____	☐ Oral ☐ IV ☐ SC	Month: __ __ Day: __ __ Year: __ __ __ __	__ __

[a]Include decimal point (if necessary) in its own box

[b]Dose unit options include: mg, mg/kg, mg/m², AUC, other; if patient's dose unit is not listed above, please write it in. If multiple dose units are available, mg is the preferred unit

[c]Route: IV=intravenous; SC=subcutaneous; if another route was used, please leave boxes blank and provide route in a comment on the Comments page

[d]If chemotherapy agent is administered over multiple days, record first date of administration

[e]Record duration of therapy (total days) for chemotherapy agent

FORM A: CHEMOTHERAPY COURSE, CYCLES, AND REGIMEN (CONT.)

Patient ID					

7. *(continued)*

ACTUAL ADMINISTRATION OF CHEMOTHERAPY
CYCLE 14

☐ Check this box if chemotherapy agent(s), dose(s), dose unit(s), route(s), and duration of therapy are exactly the same as in prior cycle and all agents in this cycle were administered on same date; then complete DATE AGENT ADMINISTERED to the right. If not, complete all fields below.

DATE AGENT ADMINISTERED:

Month		Day		Year			

	Chemotherapy Agent	Total Dose Given Per Administration[a]	Dose Unit[b]	Route[c]	Date Agent Administered[d] (Month / Day / Year)	Duration of Therapy[e] (# Days)
a.	________	☐☐☐☐☐	☐ mg ☐ mg/kg ☐ mg/m² ☐ AUC ☐ Other ______	☐ Oral ☐ IV ☐ SC	Month: ☐☐ Day: ☐☐ Year: ☐☐☐☐	☐☐
b.	________	☐☐☐☐☐	☐ mg ☐ mg/kg ☐ mg/m² ☐ AUC ☐ Other ______	☐ Oral ☐ IV ☐ SC	Month: ☐☐ Day: ☐☐ Year: ☐☐☐☐	☐☐
c.	________	☐☐☐☐☐	☐ mg ☐ mg/kg ☐ mg/m² ☐ AUC ☐ Other ______	☐ Oral ☐ IV ☐ SC	Month: ☐☐ Day: ☐☐ Year: ☐☐☐☐	☐☐
d.	________	☐☐☐☐☐	☐ mg ☐ mg/kg ☐ mg/m² ☐ AUC ☐ Other ______	☐ Oral ☐ IV ☐ SC	Month: ☐☐ Day: ☐☐ Year: ☐☐☐☐	☐☐
e.	________	☐☐☐☐☐	☐ mg ☐ mg/kg ☐ mg/m² ☐ AUC ☐ Other ______	☐ Oral ☐ IV ☐ SC	Month: ☐☐ Day: ☐☐ Year: ☐☐☐☐	☐☐

[a] Include decimal point (if necessary) in its own box
[b] Dose unit options include: mg, mg/kg, mg/m², AUC, other; if patient's dose unit is not listed above, please write it in. If multiple dose units are available, mg is the preferred unit
[c] Route: IV=intravenous; SC=subcutaneous; if another route was used, please leave boxes blank and provide route in a comment on the Comments page
[d] If chemotherapy agent is administered over multiple days, record first date of administration
[e] Record duration of therapy (total days) for chemotherapy agent

FORM A: CHEMOTHERAPY COURSE, CYCLES, AND REGIMEN (CONT.)

Patient ID					

7. *(continued)*

ACTUAL ADMINISTRATION OF CHEMOTHERAPY
CYCLE 15

☐ Check this box if chemotherapy agent(s), dose(s), dose unit(s), route(s), and duration of therapy are exactly the same as in prior cycle and all agents in this cycle were administered on same date; then complete DATE AGENT ADMINISTERED to the right. If not, complete all fields below.

DATE AGENT ADMINISTERED:

Month		Day		Year			

	Chemotherapy Agent	Total Dose Given Per Administration[a]	Dose Unit[b]	Route[c]	Date Agent Administered[d] (Month / Day / Year)	Duration of Therapy[e] # Days
a.			☐ mg ☐ mg/kg ☐ mg/m² ☐ AUC ☐ Other ______	☐ Oral ☐ IV ☐ SC		
b.			☐ mg ☐ mg/kg ☐ mg/m² ☐ AUC ☐ Other ______	☐ Oral ☐ IV ☐ SC		
c.			☐ mg ☐ mg/kg ☐ mg/m² ☐ AUC ☐ Other ______	☐ Oral ☐ IV ☐ SC		
d.			☐ mg ☐ mg/kg ☐ mg/m² ☐ AUC ☐ Other ______	☐ Oral ☐ IV ☐ SC		
e.			☐ mg ☐ mg/kg ☐ mg/m² ☐ AUC ☐ Other ______	☐ Oral ☐ IV ☐ SC		

[a]Include decimal point (if necessary) in its own box
[b]Dose unit options include: mg, mg/kg, mg/m², AUC, other; if patient's dose unit is not listed above, please write it in. If multiple dose units are available, mg is the preferred unit
[c]Route: IV=intravenous; SC=subcutaneous; if another route was used, please leave boxes blank and provide route in a comment on the Comments page
[d]If chemotherapy agent is administered over multiple days, record first date of administration
[e]Record duration of therapy (total days) for chemotherapy agent

FORM A: CHEMOTHERAPY COURSE, CYCLES, AND REGIMEN (CONT.)

Patient ID					

7. *(continued)*

ACTUAL ADMINISTRATION OF CHEMOTHERAPY
CYCLE 16

☐ Check this box if chemotherapy agent(s), dose(s), dose unit(s), route(s), and duration of therapy are exactly the same as in prior cycle and all agents in this cycle were administered on same date; then complete DATE AGENT ADMINISTERED to the right. If not, complete all fields below.

DATE AGENT ADMINISTERED:

Month		Day		Year			

	Chemotherapy Agent	Total Dose Given Per Administration[a]	Dose Unit[b]	Route[c]	Date Agent Administered[d] (Month / Day / Year)	Duration of Therapy[e] (# Days)
a.			☐ mg ☐ mg/kg ☐ mg/m² ☐ AUC ☐ Other ______	☐ Oral ☐ IV ☐ SC		
b.			☐ mg ☐ mg/kg ☐ mg/m² ☐ AUC ☐ Other ______	☐ Oral ☐ IV ☐ SC		
c.			☐ mg ☐ mg/kg ☐ mg/m² ☐ AUC ☐ Other ______	☐ Oral ☐ IV ☐ SC		
d.			☐ mg ☐ mg/kg ☐ mg/m² ☐ AUC ☐ Other ______	☐ Oral ☐ IV ☐ SC		
e.			☐ mg ☐ mg/kg ☐ mg/m² ☐ AUC ☐ Other ______	☐ Oral ☐ IV ☐ SC		

[a]Include decimal point (if necessary) in its own box
[b]Dose unit options include: mg, mg/kg, mg/m², AUC, other; if patient's dose unit is not listed above, please write it in. If multiple dose units are available, mg is the preferred unit
[c]Route: IV=intravenous; SC=subcutaneous; if another route was used, please leave boxes blank and provide route in a comment on the Comments page
[d]If chemotherapy agent is administered over multiple days, record first date of administration
[e]Record duration of therapy (total days) for chemotherapy agent

FORM A: CHEMOTHERAPY COURSE, CYCLES, AND REGIMEN (CONT.)

Patient ID					

7. *(continued)*

ACTUAL ADMINISTRATION OF CHEMOTHERAPY
CYCLE 17

☐ Check this box if chemotherapy agent(s), dose(s), dose unit(s), route(s), and duration of therapy are exactly the same as in prior cycle and all agents in this cycle were administered on same date; then complete DATE AGENT ADMINISTERED to the right. If not, complete all fields below.

DATE AGENT ADMINISTERED:

Month		Day		Year			

	Chemotherapy Agent	Total Dose Given Per Administration[a]	Dose Unit[b]	Route[c]	Date Agent Administered[d] (Month / Day / Year)	Duration of Therapy[e] # Days
a.			☐ mg ☐ mg/kg ☐ mg/m² ☐ AUC ☐ Other ______	☐ Oral ☐ IV ☐ SC	Month: __ __ Day: __ __ Year: __ __ __ __	__ __
b.			☐ mg ☐ mg/kg ☐ mg/m² ☐ AUC ☐ Other ______	☐ Oral ☐ IV ☐ SC	Month: __ __ Day: __ __ Year: __ __ __ __	__ __
c.			☐ mg ☐ mg/kg ☐ mg/m² ☐ AUC ☐ Other ______	☐ Oral ☐ IV ☐ SC	Month: __ __ Day: __ __ Year: __ __ __ __	__ __
d.			☐ mg ☐ mg/kg ☐ mg/m² ☐ AUC ☐ Other ______	☐ Oral ☐ IV ☐ SC	Month: __ __ Day: __ __ Year: __ __ __ __	__ __
e.			☐ mg ☐ mg/kg ☐ mg/m² ☐ AUC ☐ Other ______	☐ Oral ☐ IV ☐ SC	Month: __ __ Day: __ __ Year: __ __ __ __	__ __

[a]Include decimal point (if necessary) in its own box

[b]Dose unit options include: mg, mg/kg, mg/m², AUC, other; if patient's dose unit is not listed above, please write it in. If multiple dose units are available, mg is the preferred unit

[c]Route: IV=intravenous; SC=subcutaneous; if another route was used, please leave boxes blank and provide route in a comment on the Comments page

[d]If chemotherapy agent is administered over multiple days, record first date of administration

[e]Record duration of therapy (total days) for chemotherapy agent

FORM A: CHEMOTHERAPY COURSE, CYCLES, AND REGIMEN (CONT.)

Patient ID					

7. *(continued)*

ACTUAL ADMINISTRATION OF CHEMOTHERAPY
CYCLE 18

☐ Check this box if chemotherapy agent(s), dose(s), dose unit(s), route(s), and duration of therapy are exactly the same as in prior cycle and all agents in this cycle were administered on same date; then complete DATE AGENT ADMINISTERED to the right. If not, complete all fields below.

DATE AGENT ADMINISTERED:

Month		Day		Year			

	Chemotherapy Agent	Total Dose Given Per Administration[a]	Dose Unit[b]	Route[c]	Date Agent Administered[d] (Month / Day / Year)	Duration of Therapy[e] (# Days)
a.			☐ mg ☐ mg/kg ☐ mg/m² ☐ AUC ☐ Other ______	☐ Oral ☐ IV ☐ SC		
b.			☐ mg ☐ mg/kg ☐ mg/m² ☐ AUC ☐ Other ______	☐ Oral ☐ IV ☐ SC		
c.			☐ mg ☐ mg/kg ☐ mg/m² ☐ AUC ☐ Other ______	☐ Oral ☐ IV ☐ SC		
d.			☐ mg ☐ mg/kg ☐ mg/m² ☐ AUC ☐ Other ______	☐ Oral ☐ IV ☐ SC		
e.			☐ mg ☐ mg/kg ☐ mg/m² ☐ AUC ☐ Other ______	☐ Oral ☐ IV ☐ SC		

[a]Include decimal point (if necessary) in its own box

[b]Dose unit options include: mg, mg/kg, mg/m², AUC, other; if patient's dose unit is not listed above, please write it in. If multiple dose units are available, mg is the preferred unit

[c]Route: IV=intravenous; SC=subcutaneous; if another route was used, please leave boxes blank and provide route in a comment on the Comments page

[d]If chemotherapy agent is administered over multiple days, record first date of administration

[e]Record duration of therapy (total days) for chemotherapy agent

FORM A: FN RISK FACTORS AND OTHER VARIABLES

Patient ID					

8. Please record the patient's age *OR* birth date on the TREATMENT INITIATION DATE. If patient age on the TREATMENT INITIATION DATE is greater than the maximum reportable age, record age using greater than sign (>) before maximum reportable age (in its own box).

AGE: ☐☐☐ ***OR*** **BIRTH DATE:**

Month		Day		Year			

9. What is the patient's sex?

☐ Male ☐ Female

10. Please specify as much anthropometric information as is available about the patient. Use the most proximate values for height, weight, body mass index (BMI), and body surface area (BSA) within 1 year prior to the TREATMENT INITIATION DATE. Please include the date of measurement. Anthropometric information from chemotherapy order notes may be used in lieu of other sources of such information (even if other information was collected more proximate to the TREATMENT INITIATION DATE).

	Height[a]				Height Unit			
**Value:**					☐ inches ☐ centimeters			
	**Month**		**Day**		**Year**			
**Date:**								

[a]Record as inches or centimeters; round to whole number

Weight[a]				Weight Unit			
				☐ pounds ☐ kilograms			
**Month**		**Day**		**Year**			

[a]Record as pounds or kilograms; round to whole number

	BMI[a]							
**Value:**								
	**Month**		**Day**		**Year**			
**Date:**								

[a]Round to first digit to right of decimal point

BSA[a]							
**Month**		**Day**		**Year**			

[a]Round to first digit to right of decimal point

FORM A: FN RISK FACTORS AND OTHER VARIABLES (CONT.)

Patient ID					

11. Please report lab values and units for the following measures (most proximate value within 180 days on or before the TREATMENT INITIATION DATE). If a lab value includes a greater than or less than sign (> or <), please include it in its own box.

	Lab Value	Lab Unit
**Blood Glucose:**		mg/dL
**HbA1c:**		%
**Bilirubin:**		mg/dL
**ALT:**		☐ U/L<br>☐ IU/L
**AST:**		☐ U/L<br>☐ IU/L
**ANC:**		☐ x 10^9/L<br>☐ x 1000/µL<br>☐ cells/µL<br>☐ x K/µL<br>☐ x K/CUMM

	Lab Value	Lab Unit
**GFR:**		mL/min
**Creatinine:**		mg/dL
**Hemoglobin:**		☐ g/dL<br>☐ mg/dL
**Albumin:**		g/dL
**ALP:**		☐ U/L<br>☐ IU/L
**ALC:**		☐ x 10^9/L<br>☐ x 1000/µL<br>☐ cells/µL<br>☐ x K/µL<br>☐ x K/CUMM
**LDH:**		☐ U/L<br>☐ IU/L

ALC=Absolute lymphocyte count; ALP=Alkaline phosphatase; ALT=Alanine transaminase; ANC= Absolute neutrophil count; AST=Aspartate aminotransferase; GFR=Glomerular filtration rate; HbA1c=Glycated hemoglobin; LDH=Lactate dehydrogenase

12. Does the patient have a history of any of the following comorbidities within 1 year prior to the TREATMENT INITIATION DATE? Please select all that apply.

- ☐ Cardiovascular Disease (CVD)
  - ☐ Ischemic Heart Disease
  - ☐ Cerebrovascular Disease
  - ☐ Heart Failure
  - ☐ Cardiac Dysrhythmias
- ☐ Diabetes
- ☐ Gastrointestinal Ulcers
- ☐ HIV/AIDS
- ☐ Kidney Disease (Chronic)
- ☐ Liver Disease
- ☐ Lung Disease
  - ☐ Chronic Obstructive Pulmonary Disease (COPD)
    - ☐ Chronic Bronchitis
    - ☐ Emphysema
    - ☐ Not Otherwise Specified
  - ☐ Asthma
  - ☐ Other Lung Disease
- ☐ Lupus/Systemic Lupus
- ☐ Malnutrition
- ☐ Multiple Sclerosis
- ☐ Osteoarthrosis/Spondylosis
- ☐ Regional Enteritis (e.g., Crohn's Disease, Colitis)
- ☐ Rheumatoid Disease
- ☐ Thyroid Disorder
- ☐ None of the Above

FORM A: FN RISK FACTORS AND OTHER VARIABLES (CONT.)

Patient ID					

13. Does the patient have a pre-existing condition (within 90 days on or before the TREATMENT INITIATION DATE)? Please select all that apply.

☐ Neutropenia ☐ Anemia ☐ Infection ☐ None

14. If performance status was evaluated prior to initiation of chemotherapy, please specify measure (e.g., ECOG, Karnofsky), value, and date evaluated (most proximate value within 1 year on or prior to the TREATMENT INITIATION DATE). If performance status was not documented but can be evaluated from information in the Medical Chart, please specify measure used, corresponding value, and date from which the evidence for performance status was ascertained. Please use the comment section below to state if performance status was assigned retrospectively or to provide any other information. (If more space is required, continue writing in the comment section on the last page of Form A.)

**Measure:**	☐ ECOG/Zubrod ☐ Karnofsky<br>☐ Other ______________

☐ None Found

	**Month**		**Day**		**Year**			
**Date Evaluated:**								

**Value:**				

**Comment:**	--

15. Did the patient have metastatic disease on the TREATMENT INITIATION DATE?

☐ No

☐ Yes *If yes, was the metastasis to bone?*

☐ No

☐ Yes

16. Did the patient receive an erythropoietin stimulating agent (ESA)?

☐ No

☐ Yes *If yes, specify the first agent received and select the cycles for which **ANY ESA** was received (only include administration through the 18th cycle):*

**Agent:**																		

**Cycle(s):**	☐ 1 ☐ 2 ☐ 3 ☐ 4 ☐ 5 ☐ 6 ☐ 7 ☐ 8 ☐ 9<br>☐ 10 ☐ 11 ☐ 12 ☐ 13 ☐ 14 ☐ 15 ☐ 16 ☐ 17 ☐ 18

FORM A: FN RISK FACTORS AND OTHER VARIABLES (CONT.)

Patient ID					

17. Does the patient have a history of receipt of myelosuppressive agents at any time prior to the TREATMENT INITIATION DATE?

☐ No

☐ Yes *If yes, provide date of the last administration of myelosuppressive agent:*

Month		Day		Year			

If yes, what was reason for administration of myelosuppressive agent?

☐ Cancer

☐ Other reason

18. a) Does the patient have a history of radiation therapy at any time prior to the TREATMENT INITIATION DATE?

☐ No

☐ Yes *If yes, provide the following information related to the patient's last round of radiation therapy (most proximate to the TREATMENT INITIATION DATE):*

	Month		Day		Year			
**Date Started:**								

	Month		Day		Year			
**Date Stopped:**								

**Total Dose (Cumulative):**				

**Dose Unit:**	☐ cGy ☐ Gy ☐ Other ______________

**Site:**																

b) Did the patient receive radiation therapy at any time DURING the INDEX COURSE?

☐ No

☐ Yes *If yes, provide date of the first administration of radiation therapy (most proximate to the TREATMENT INITIATION DATE):*

	Month		Day		Year			
**Date Started:**								

19. Did the patient undergo one of the selected surgical procedures during the 60 days prior to the TREATMENT INITIATION DATE?

☐ No ☐ Yes

FORM A: FN RISK FACTORS AND OTHER VARIABLES (CONT.)

Patient ID					

20. a) Did the patient receive glucocorticosteroids (oral or IV) during the **30** days on or prior to the TREATMENT INITIATION DATE?

☐ No

☐ Yes *If yes, in the table below, provide the agent name, dose, dose unit, and days supplied/administered for each instance an agent was received during the **180** days on or prior to the TREATMENT INITIATION DATE.*

b) Did the patient receive any immunosuppressive agents other than glucocorticosteroids during the **90** days on or prior to the TREATMENT INITIATION DATE?

☐ No

☐ Yes *If yes, in the table below, provide the agent name, dose, dose unit, and days supplied/administered for each instance an agent was received during the **90** days on or prior to the TREATMENT INITIATION DATE.*

c)

	Immunosuppressive Agent	Total Dose Given Per Administration[a]				Dose Unit[b]	Number of Days Supplied or Administered		
I)						☐ mg ☐ mL ☐ mcg<br>☐ Other ____________			
II)						☐ mg ☐ mL ☐ mcg<br>☐ Other ____________			
III)						☐ mg ☐ mL ☐ mcg<br>☐ Other ____________			
IV)						☐ mg ☐ mL ☐ mcg<br>☐ Other ____________			
V)						☐ mg ☐ mL ☐ mcg<br>☐ Other ____________			

[a]Include decimal point (if necessary) in its own box

[b]Dose unit options include: mg, mL, mcg, other; if patient's dose unit is not listed above, please write it in

FORM A: OTHER OUTCOMES

Patient ID					

21. Was the patient hospitalized for *ANY* reason during the INDEX COURSE?

☐ No

☐ Yes *If yes, record the dates of admission and discharge during the INDEX COURSE. If more than 5 hospitalizations occurred, only record the first 5 hospitalizations:*

	Date of Admission								Date of Discharge							
	Month		Day		Year				Month		Day		Year			
a.																
b.																
c.																
d.																
e.																

22. Did the patient die during the INDEX COURSE (i.e., from the TREATMENT INITIATION DATE through 35 days after first administration of chemotherapy in the last cycle)?

☐ No

☐ Yes *If yes, please provide date of death:*

Month		Day		Year			

FORM A: COMMENTS

Patient ID					

Specify Form & Question Number	Comments for All Forms (A, B, C & D) (If Necessary)

Date Form Completed		
Month	Day	Year

Reviewer ID

Patient ID

FORM B: FEBRILE NEUTROPENIA

Please record ***ALL*** *evidence of FN for the entire chemotherapy course (i.e., from the TREATMENT INITIATION DATE through the last cycle of chemotherapy [up to 18]).*

1. Please indicate every instance in which the patient's absolute neutrophil count (ANC) was <1.0 x 10^9/L or <1.0 x 1000/µL or <1000 cells/µL or <1.0 x K/µL or <1.0 x K/CUMM *AND* there was evidence of ≥1 other variable (as listed in the table below). Please record all supporting evidence regardless of care setting.

	Date of Evidence			ANC Value[a]	ANC Value Unit	Body Temperature ≥38.3 °C (101 °F)	Infection Diagnosis	IV AMB	Oral AMB
	Month	Day	Year						
a.					☐ x 10^9/L ☐ x 1000/µL ☐ cells/µL ☐ x K/µL ☐ x K/CUMM	☐ Same day ☐ ± 1 day	☐ Same day ☐ ± 1 day	☐ Same day ☐ ± 1 day	☐ Same day ☐ ± 1 day
b.					☐ x 10^9/L ☐ x 1000/µL ☐ cells/µL ☐ x K/µL ☐ x K/CUMM	☐ Same day ☐ ± 1 day	☐ Same day ☐ ± 1 day	☐ Same day ☐ ± 1 day	☐ Same day ☐ ± 1 day
c.					☐ x 10^9/L ☐ x 1000/µL ☐ cells/µL ☐ x K/µL ☐ x K/CUMM	☐ Same day ☐ ± 1 day	☐ Same day ☐ ± 1 day	☐ Same day ☐ ± 1 day	☐ Same day ☐ ± 1 day
d.					☐ x 10^9/L ☐ x 1000/µL ☐ cells/µL ☐ x K/µL ☐ x K/CUMM	☐ Same day ☐ ± 1 day	☐ Same day ☐ ± 1 day	☐ Same day ☐ ± 1 day	☐ Same day ☐ ± 1 day
e.					☐ x 10^9/L ☐ x 1000/µL ☐ cells/µL ☐ x K/µL ☐ x K/CUMM	☐ Same day ☐ ± 1 day	☐ Same day ☐ ± 1 day	☐ Same day ☐ ± 1 day	☐ Same day ☐ ± 1 day
f.					☐ x 10^9/L ☐ x 1000/µL ☐ cells/µL ☐ x K/µL ☐ x K/CUMM	☐ Same day ☐ ± 1 day	☐ Same day ☐ ± 1 day	☐ Same day ☐ ± 1 day	☐ Same day ☐ ± 1 day

[a]Include decimal point (if necessary) in its own box

FORM B: FEBRILE NEUTROPENIA (CONT.)

Patient ID					

Please record ALL evidence of FN for the entire chemotherapy course (i.e., from the TREATMENT INITIATION DATE through the last cycle of chemotherapy [up to 18]).

1. ***(Continued)*** Please indicate every instance in which the patient's absolute neutrophil count (ANC) was <1.0 x 10^9/L or <1.0 x 1000/μL or <1000 cells/μL or <1.0 x K/μL or <1.0 x K/CUMM AND there was evidence of ≥1 other variable (as listed in the table below). Please record all supporting evidence regardless of care setting.

	Date of Evidence			ANC Value[a]	ANC Value Unit	Body Temperature ≥38.3 ^{0}C (101 ^{0}F)	Infection Diagnosis	IV AMB	Oral AMB
	Month	Day	Year						
g.					☐ x 10^9/L ☐ x 1000/μL ☐ cells/μL ☐ x K/μL ☐ x K/CUMM	☐ Same day ☐ ± 1 day	☐ Same day ☐ ± 1 day	☐ Same day ☐ ± 1 day	☐ Same day ☐ ± 1 day
h.					☐ x 10^9/L ☐ x 1000/μL ☐ cells/μL ☐ x K/μL ☐ x K/CUMM	☐ Same day ☐ ± 1 day	☐ Same day ☐ ± 1 day	☐ Same day ☐ ± 1 day	☐ Same day ☐ ± 1 day
i.					☐ x 10^9/L ☐ x 1000/μL ☐ cells/μL ☐ x K/μL ☐ x K/CUMM	☐ Same day ☐ ± 1 day	☐ Same day ☐ ± 1 day	☐ Same day ☐ ± 1 day	☐ Same day ☐ ± 1 day
j.					☐ x 10^9/L ☐ x 1000/μL ☐ cells/μL ☐ x K/μL ☐ x K/CUMM	☐ Same day ☐ ± 1 day	☐ Same day ☐ ± 1 day	☐ Same day ☐ ± 1 day	☐ Same day ☐ ± 1 day
k.					☐ x 10^9/L ☐ x 1000/μL ☐ cells/μL ☐ x K/μL ☐ x K/CUMM	☐ Same day ☐ ± 1 day	☐ Same day ☐ ± 1 day	☐ Same day ☐ ± 1 day	☐ Same day ☐ ± 1 day
l.					☐ x 10^9/L ☐ x 1000/μL ☐ cells/μL ☐ x K/μL ☐ x K/CUMM	☐ Same day ☐ ± 1 day	☐ Same day ☐ ± 1 day	☐ Same day ☐ ± 1 day	☐ Same day ☐ ± 1 day

[a]Include decimal point (if necessary) in its own box

FORM B: FEBRILE NEUTROPENIA (CONT.)

Patient ID					

Please record ALL evidence of FN for the entire chemotherapy course (i.e., from the TREATMENT INITIATION DATE through the last cycle of chemotherapy [up to 18]).

1. ***(Continued)*** Please indicate every instance in which the patient's absolute neutrophil count (ANC) was <1.0 x 10^9/L or <1.0 x 1000/µL or <1000 cells/µL or <1.0 x K/µL or <1.0 x K/CUMM AND there was evidence of ≥1 other variable (as listed in the table below). Please record all supporting evidence regardless of care setting.

	Date of Evidence (Month / Day / Year)	ANC Value[a]	ANC Value Unit	Body Temperature ≥38.3 °C (101 °F)	Infection Diagnosis	IV AMB	Oral AMB
m.	Month ☐☐ Day ☐☐ Year ☐☐☐☐	☐☐☐☐	☐ x 10^9/L ☐ x 1000/µL ☐ cells/µL ☐ x K/µL ☐ x K/CUMM	☐ Same day ☐ ± 1 day	☐ Same day ☐ ± 1 day	☐ Same day ☐ ± 1 day	☐ Same day ☐ ± 1 day
n.	Month ☐☐ Day ☐☐ Year ☐☐☐☐	☐☐☐☐	☐ x 10^9/L ☐ x 1000/µL ☐ cells/µL ☐ x K/µL ☐ x K/CUMM	☐ Same day ☐ ± 1 day	☐ Same day ☐ ± 1 day	☐ Same day ☐ ± 1 day	☐ Same day ☐ ± 1 day
o.	Month ☐☐ Day ☐☐ Year ☐☐☐☐	☐☐☐☐	☐ x 10^9/L ☐ x 1000/µL ☐ cells/µL ☐ x K/µL ☐ x K/CUMM	☐ Same day ☐ ± 1 day	☐ Same day ☐ ± 1 day	☐ Same day ☐ ± 1 day	☐ Same day ☐ ± 1 day
p.	Month ☐☐ Day ☐☐ Year ☐☐☐☐	☐☐☐☐	☐ x 10^9/L ☐ x 1000/µL ☐ cells/µL ☐ x K/µL ☐ x K/CUMM	☐ Same day ☐ ± 1 day	☐ Same day ☐ ± 1 day	☐ Same day ☐ ± 1 day	☐ Same day ☐ ± 1 day
q.	Month ☐☐ Day ☐☐ Year ☐☐☐☐	☐☐☐☐	☐ x 10^9/L ☐ x 1000/µL ☐ cells/µL ☐ x K/µL ☐ x K/CUMM	☐ Same day ☐ ± 1 day	☐ Same day ☐ ± 1 day	☐ Same day ☐ ± 1 day	☐ Same day ☐ ± 1 day
r.	Month ☐☐ Day ☐☐ Year ☐☐☐☐	☐☐☐☐	☐ x 10^9/L ☐ x 1000/µL ☐ cells/µL ☐ x K/µL ☐ x K/CUMM	☐ Same day ☐ ± 1 day	☐ Same day ☐ ± 1 day	☐ Same day ☐ ± 1 day	☐ Same day ☐ ± 1 day

[a]Include decimal point (if necessary) in its own box

FORM B: FEBRILE NEUTROPENIA (CONT.)

Patient ID					

2. Please indicate every instance in which there was a diagnosis of neutropenia, and/or fever, and/or infection during the chemotherapy course. Please specify the date of encounter/admission, the setting of care in which the diagnosis was recorded, and whether the patient received any antimicrobials (AMBs). If multiple diagnoses are recorded on the same day but in different settings of care, please record on separate rows.

	Date of Encounter/Date of Admission			Diagnosis	Setting of Care	IV AMB	Oral AMB
	Month	Day	Year				
a.				☐ Neutropenia<br>☐ Fever<br>☐ Infection	☐ Inpatient<br>☐ Outpatient	☐ Same day<br>☐ ± 1 day	☐ Same day<br>☐ ± 1 day
b.				☐ Neutropenia<br>☐ Fever<br>☐ Infection	☐ Inpatient<br>☐ Outpatient	☐ Same day<br>☐ ± 1 day	☐ Same day<br>☐ ± 1 day
c.				☐ Neutropenia<br>☐ Fever<br>☐ Infection	☐ Inpatient<br>☐ Outpatient	☐ Same day<br>☐ ± 1 day	☐ Same day<br>☐ ± 1 day
d.				☐ Neutropenia<br>☐ Fever<br>☐ Infection	☐ Inpatient<br>☐ Outpatient	☐ Same day<br>☐ ± 1 day	☐ Same day<br>☐ ± 1 day
e.				☐ Neutropenia<br>☐ Fever<br>☐ Infection	☐ Inpatient<br>☐ Outpatient	☐ Same day<br>☐ ± 1 day	☐ Same day<br>☐ ± 1 day
f.				☐ Neutropenia<br>☐ Fever<br>☐ Infection	☐ Inpatient<br>☐ Outpatient	☐ Same day<br>☐ ± 1 day	☐ Same day<br>☐ ± 1 day
g.				☐ Neutropenia<br>☐ Fever<br>☐ Infection	☐ Inpatient<br>☐ Outpatient	☐ Same day<br>☐ ± 1 day	☐ Same day<br>☐ ± 1 day
h.				☐ Neutropenia<br>☐ Fever<br>☐ Infection	☐ Inpatient<br>☐ Outpatient	☐ Same day<br>☐ ± 1 day	☐ Same day<br>☐ ± 1 day
i.				☐ Neutropenia<br>☐ Fever<br>☐ Infection	☐ Inpatient<br>☐ Outpatient	☐ Same day<br>☐ ± 1 day	☐ Same day<br>☐ ± 1 day
j.				☐ Neutropenia<br>☐ Fever<br>☐ Infection	☐ Inpatient<br>☐ Outpatient	☐ Same day<br>☐ ± 1 day	☐ Same day<br>☐ ± 1 day

Date Form Completed		
Month	Day	Year

Reviewer ID

Patient ID

FORM C: COLONY-STIMULATING FACTOR (CSF) USE

1. Please provide information related to use of colony-stimulating factors (CSF) in each cycle of chemotherapy (up to 18 cycles). Please describe each course of administration on a separate line; if the dose of a daily agent changed during the course of administration, please use a separate line to record each dose. If use of a CSF agent spanned two or more consecutive days, please record on only one line. Please capture pharmacy dispenses of CSF agents during the 7-day window prior to the beginning of each cycle. ***NOTE: Please complete additional copies of this form as needed, and fill in the appropriate page numbers above.***

	CSF Agent	Dose Administered[a]	Dose Unit	Route[b]	Start Date			Stop Date			Duration of Therapy[c]
					Month	Day	Year	Month	Day	Year	# Days
a.	☐ Pegfilgrastim ☐ tbo-filgrastim ☐ Filgrastim ☐ Sargramostim ☐ Pegfilgrastim Biosimilar ☐ Filgrastim Biosimilar		☐ mg ☐ mcg	☐ PFS/MDV ☐ OBI							
b.	☐ Pegfilgrastim ☐ tbo-filgrastim ☐ Filgrastim ☐ Sargramostim ☐ Pegfilgrastim Biosimilar ☐ Filgrastim Biosimilar		☐ mg ☐ mcg	☐ PFS/MDV ☐ OBI							
c.	☐ Pegfilgrastim ☐ tbo-filgrastim ☐ Filgrastim ☐ Sargramostim ☐ Pegfilgrastim Biosimilar ☐ Filgrastim Biosimilar		☐ mg ☐ mcg	☐ PFS/MDV ☐ OBI							
d.	☐ Pegfilgrastim ☐ tbo-filgrastim ☐ Filgrastim ☐ Sargramostim ☐ Pegfilgrastim Biosimilar ☐ Filgrastim Biosimilar		☐ mg ☐ mcg	☐ PFS/MDV ☐ OBI							
e.	☐ Pegfilgrastim ☐ tbo-filgrastim ☐ Filgrastim ☐ Sargramostim ☐ Pegfilgrastim Biosimilar ☐ Filgrastim Biosimilar		☐ mg ☐ mcg	☐ PFS/MDV ☐ OBI							
f.	☐ Pegfilgrastim ☐ tbo-filgrastim ☐ Filgrastim ☐ Sargramostim ☐ Pegfilgrastim Biosimilar ☐ Filgrastim Biosimilar		☐ mg ☐ mcg	☐ PFS/MDV ☐ OBI							

[a] For pegfilgrastim, record total dose administered; for daily agents, record daily dose administered

[b] Route of administration: pre-filled syringe (PFS)/multi-dose vial (MDV) or on-body injector (OBI)

[c] Record duration of therapy (total days) for CSF agent; if days were skipped, please record explanation on Form A Comments page

Date Form Completed		
Month	Day	Year

Reviewer ID

Patient ID

FORM D: ANTIMICROBIAL (AMB) PROPHYLAXIS

1. Please provide information related to antimicrobial (AMB) prophylaxis. If AMB agent, dose, dose unit, route of administration, and duration of therapy are exactly the same in multiple cycles, please include on one line with the appropriate "cycles received" marked. If any of these variables differ between cycles, please complete separate lines. Only include administration from the first day of myelosuppressive chemotherapy administration through the 5th day following completion of myelosuppressive chemotherapy administration (per cycle, up to 18 cycles). Please capture pharmacy dispenses of AMB agents during the 7-day window prior to the beginning of each cycle. ***NOTE: Please complete additional copies of this form as needed, and fill in the appropriate page numbers above.***

	AMB Agent	Dose Administered[a]	Dose Unit[b]	Route	Duration of Therapy Per Cycle[c]	Cycles Received (Mark each cycle in which AMB prophylaxis was received)
a.	----------		☐ gm ☐ mg ☐ IU ☐ mg/kg ☐ mcg ☐ Other ______	☐ Oral ☐ Other ☐ IV ☐ Unknown		☐ 1 ☐ 2 ☐ 3 ☐ 4 ☐ 5 ☐ 6 ☐ 7 ☐ 8 ☐ 9 ☐ 10 ☐ 11 ☐ 12 ☐ 13 ☐ 14 ☐ 15 ☐ 16 ☐ 17 ☐ 18
b.	----------		☐ gm ☐ mg ☐ IU ☐ mg/kg ☐ mcg ☐ Other ______	☐ Oral ☐ Other ☐ IV ☐ Unknown		☐ 1 ☐ 2 ☐ 3 ☐ 4 ☐ 5 ☐ 6 ☐ 7 ☐ 8 ☐ 9 ☐ 10 ☐ 11 ☐ 12 ☐ 13 ☐ 14 ☐ 15 ☐ 16 ☐ 17 ☐ 18
c.	----------		☐ gm ☐ mg ☐ IU ☐ mg/kg ☐ mcg ☐ Other ______	☐ Oral ☐ Other ☐ IV ☐ Unknown		☐ 1 ☐ 2 ☐ 3 ☐ 4 ☐ 5 ☐ 6 ☐ 7 ☐ 8 ☐ 9 ☐ 10 ☐ 11 ☐ 12 ☐ 13 ☐ 14 ☐ 15 ☐ 16 ☐ 17 ☐ 18
d.	----------		☐ gm ☐ mg ☐ IU ☐ mg/kg ☐ mcg ☐ Other ______	☐ Oral ☐ Other ☐ IV ☐ Unknown		☐ 1 ☐ 2 ☐ 3 ☐ 4 ☐ 5 ☐ 6 ☐ 7 ☐ 8 ☐ 9 ☐ 10 ☐ 11 ☐ 12 ☐ 13 ☐ 14 ☐ 15 ☐ 16 ☐ 17 ☐ 18
e.	----------		☐ gm ☐ mg ☐ IU ☐ mg/kg ☐ mcg ☐ Other ______	☐ Oral ☐ Other ☐ IV ☐ Unknown		☐ 1 ☐ 2 ☐ 3 ☐ 4 ☐ 5 ☐ 6 ☐ 7 ☐ 8 ☐ 9 ☐ 10 ☐ 11 ☐ 12 ☐ 13 ☐ 14 ☐ 15 ☐ 16 ☐ 17 ☐ 18

[a]For IV AMBs, record total dose administered; for oral AMBs, record daily dose administered

[b]Dose unit options include: gm, mg, IU, mg/kg, mcg, other; if patient's dose unit is not listed above, please write it in

[c]Record duration of therapy (total days per cycle) for oral and IV AMBs

Table S1. Baseline Demographics, Characteristics, and Comorbidities of Patients With Metastatic Cancer Who Did Not Receive G-CSF Prophylaxis in Cycle 1 Stratified by Cancer Type and Chemotherapy FN Risk Level

	**All Cancers**			**Breast Cancer**			**Colorectal Cancer**			**Lung Cancer**			**NHL**		
	**High Risk (n=154)**	**IR + ≥1 Risk Factor (n=352)**	**All others[a] (n=666)**	**High Risk (n=34)**	**IR + ≥1 Risk Factor (n=29)**	**All others[a] (n=134)**	**High Risk (n=0)**	**IR + ≥1 Risk Factor (n=173)**	**All others[a] (n=179)**	**High Risk (n=107)**	**IR + ≥1 Risk Factor (n=132)**	**All others[a] (n=324)**	**High Risk (n=13)**	**IR + ≥1 Risk Factor (n=18)**	**All others[a] (n=29)**
Patient															
Age, mean (SD), years	66.2 (12.3)	62.5 (11.9)	64.8 (11.7)	57.8 (11.7)	59.4 (11.5)	59.7 (12.5)	–	59.6 (12.5)	64.2 (13.4)	68.3 (11.5)	66.6 (9.8)	67.3 (9.6)	70.8 (11.4)	65.7 (11.0)	64.1 (10.5)
Men, %	46.1	42.6	41.0	0	0	0	–	45.1	48.6	57.0	47.7	51.5	76.9	50.0	65.5
BMI, mean (SD), kg/m^2	27.3 (6.5)	28.8 (7.2)	28.7 (6.8)	28.4 (6.2)	29.3 (9.7)	30.7 (6.6)	–	29.4 (6.8)	29.4 (7.3)	26.9 (6.7)	27.6 (6.9)	27.3 (6.0)	28.2 (6.0)	30.9 (7.2)	31.1 (8.3)
Laboratory values, mean (SD)[b]															
Albumin, g/dL	3.8 (0.6)	3.9 (0.6)	3.8 (0.5)	4.1 (0.5)	4.1 (0.6)	3.9 (0.6)	–	3.9 (0.5)	3.8 (0.5)	3.7 (0.6)	3.9 (0.6)	3.7 (0.5)	4.0 (0.7)	3.9 (0.5)	3.9 (0.4)
ALC, K/µL	1.5 (1.7)	1.6 (1.2)	1.5 (0.9)	1.8 (0.8)	1.8 (0.7)	1.5 (0.7)	–	1.6 (0.7)	1.6 (0.7)	1.3 (0.6)	1.6 (1.7)	1.4 (0.8)	2.6 (5.2)	1.9 (1.3)	2.1 (2.3)
ALP, U/L	101.5 (59.0)	114.1 (104.8)	118.1 (115.6)	79.8 (23.7)	129.4 (145.7)	130.6 (138.5)	–	115.8 (118.6)	141.8 (157.8)	111.1 (67.5)	111.3 (76.6)	102.4 (70.1)	85.5 (29.7)	94.9 (30.1)	84.7 (23.8)
ALT, U/L	27.0 (20.1)	32.4 (35.2)	28.6 (26.7)	26.3 (17.6)	34.6 (45.3)	34.2 (37.6)	–	32.2 (26.5)	30.1 (29.1)	28.0 (21.5)	33.0 (44.7)	25.6 (18.6)	19.4 (8.8)	24.9 (15.9)	28.8 (26.8)
ANC, K/µL	7.0 (3.9)	5.5 (2.7)	6.2 (3.5)	5.5 (2.8)	5.2 (2.1)	5.3 (4.0)	–	5.0 (2.4)	5.6 (2.8)	7.5 (4.0)	6.4 (3.0)	7.0 (3.5)	5.8 (4.7)	5.0 (2.1)	5.2 (3.6)
AST, U/L	27.0 (36.5)	35.3 (30.5)	32.2 (40.5)	23.1 (11.8)	42.7 (47.9)	44.7 (72.5)	–	33.6 (25.8)	37.4 (40.3)	28.5 (43.6)	36.9 (33.3)	24.9 (14.7)	25.5 (9.0)	28.3 (12.7)	24.8 (9.9)
Bilirubin, mg/dL	0.5 (0.2)	0.5 (0.4)	0.6 (1.4)	0.4 (0.2)	0.5 (0.2)	0.6 (1.0)	–	0.5 (0.3)	0.8 (2.6)	0.5 (0.2)	0.6 (0.4)	0.5 (0.2)	0.6 (0.2)	0.4 (0.2)	0.5 (0.2)
Blood glucose, mg/dL	123.3 (56.4)	113.0 (39.4)	116.3 (46.0)	108.2 (45.4)	116.8 (50.7)	114.4 (49.9)	–	110.4 (37.5)	114.8 (43.2)	128.1 (59.6)	111.1 (31.2)	118.2 (46.5)	122.6 (51.2)	148.3 (70.1)	111.8 (43.9)
GFR, mL/min	73.5 (20.5)	77.2 (21.0)	75.6 (22.5)	68.5 (20.3)	70.5 (20.7)	79.1 (23.5)	–	79.1 (21.4)	74.8 (23.2)	76.2 (20.7)	78.1 (20.7)	74.8 (21.6)	64.2 (14.4)	64.4 (13.3)	73.7 (23.3)
Hemoglobin, g/dL	12.3 (1.7)	12.6 (2.3)	12.5 (1.8)	13.0 (1.5)	14.1 (5.5)	12.4 (1.8)	–	12.0 (1.7)	12.0 (1.8)	12.0 (1.6)	13.1 (1.6)	12.7 (1.7)	13.0 (1.8)	12.5 (1.9)	12.4 (2.0)
Chronic comorbidities, %[c]															
Cardiovascular disease[d]	25.3	20.2	24.0	2.9	0	12.7	–	16.2	25.1	29.0	27.3	27.8	53.8	38.9	27.6
Diabetes	22.7	18.8	22.7	11.8	17.2	14.9	–	19.7	26.8	24.3	18.2	23.8	38.5	16.7	20.7
Kidney disease (chronic)	5.8	8.5	10.4	0	3.4	7.5	–	7.5	10.1	6.5	9.8	11.7	15.4	16.7	10.3
Lung disease[e]	44.8	24.7	28.7	17.6	13.8	17.9	–	11.6	12.8	56.1	44.7	42.9	23.1	22.2	17.2
Osteoarthrosis/spondylosis	16.2	14.8	17.4	17.6	0	17.2	–	14.5	12.3	15.9	14.4	17.9	15.4	44.4	44.8
Thyroid disorder	13.6	14.8	12.8	23.5	13.8	12.7	–	15.0	14.5	9.3	15.2	12.0	23.1	11.1	10.3
Pre-existing conditions, %															
Neutropenia	0.6	2.0	2.3	0	6.9	1.5	–	0.6	3.9	0	3.0	1.5	7.7	0	3.4
Anemia	24.7	23.9	20.6	2.9	3.4	14.2	–	32.4	31.8	32.7	20.5	17.3	15.4	0	17.2
Infection	24.7	26.4	18.0	17.6	34.5	9.7	–	20.8	22.9	27.1	31.8	17.9	23.1	27.8	27.6
History of myelosuppressive cancer treatment, %	9.7	13.1	30.6	11.8	24.1	52.2	–	13.9	30.7	8.4	11.4	21.6	15.4	0	31.0
History of radiation therapy, %	29.9	18.5	33.8	8.8	17.2	47.0	–	10.4	14.0	38.3	31.1	41.0	15.4	5.6	13.8
Radiation during the course, %	6.5	1.7	3.6	26.5	0	7.5	–	1.7	0	0.9	2.3	4.3	0	0	0
Surgery before treatment initiation date, %	20.8	41.5	19.8	47.1	34.5	17.9	–	59.5	26.8	11.2	22.0	17.9	30.8	22.2	6.9
Receipt of immunosuppressive agents, %															
≥90 days glucocorticoids	87.7	92.9	81.2	82.4	86.2	67.2	–	93.1	75.4	90.7	96.2	90.4	76.9	77.8	79.3
Other immunosuppressive agents	0	0.3	1.2	0	0	0.7	–	0	0	0	0.8	2.2	0	0	0
Cancer															
Metastases to bone, %	22.1	15.6	24.5	11.8	20.7	51.5	–	2.3	2.8	26.2	31.8	26.5	15.4	16.7	10.3

ALC: absolute lymphocyte count; ALP: alkaline phosphatase; ALT: alanine transaminase; ANC: absolute neutrophil count; AST: aspartate aminotransferase; BMI: body mass index; G-CSF: granulocyte colony-stimulating factor; GFR: glomerular filtration rate; IR: intermediate risk; FN: febrile neutropenia; G-CSF: granulocyte colony-stimulating factor; LR: low risk; NHL: non-Hodgkin lymphoma; UR: unknown risk.

[a]IR Chemotherapy + 0 Risk Factors or LR/UR Chemotherapy.

[b]Laboratory values not available for all patients.

[c]Results presented for comorbidities among >5% of study population.

[d]Cardiovascular disease includes ischemic heart disease, cerebrovascular disease, heart failure, and cardiac dysrhythmias.

[e]Lung disease includes chronic obstructive pulmonary disease, asthma, and other lung diseases.

Table S2. Frequency of Most Common Planned Chemotherapy Regimens Among Patients With Metastatic Cancer[a]

Metastatic Cancer/Chemotherapy Regimen	n (%)	Periodicity (%)					
		QW	Q2W	Q3W	Q4W	Unknown	Other
Breast cancer							
Cyclophosphamide + doxorubicin + paclitaxel ± trastuzumab	65 (17.1)	0	75.4	24.6	0	0	0
Cyclophosphamide + doxorubicin	40 (10.5)	0	65.0	32.5	2.5	0	0
Cyclophosphamide + docetaxel	39 (10.3)	0	0	100	0	0	0
Paclitaxel	31 (8.2)	3.2	0	19.4	77.4	0	0
Cyclophosphamide + docetaxel + doxorubicin	22 (5.8)	0	22.7	72.7	4.5	0	0
Colorectal cancer							
5-Fluorouracil + 5-fluorouracil (continuous infusion) + leucovorin + oxaliplatin	129 (35.8)	0	100	0	0	0	0
5-Fluorouracil + 5-fluorouracil (continuous infusion) + bevacizumab + leucovorin + oxaliplatin	68 (18.9)	0	98.5	1.5	0	0	0
Capecitabine + oxaliplatin	36 (10.0)	0	11.1	88.9	0	0	0
5-Fluorouracil + 5-fluorouracil (continuous infusion) + bevacizumab + irinotecan + leucovorin	16 (4.4)	0	100	0	0	0	0
5-Fluorouracil + 5-fluorouracil (continuous infusion) + irinotecan + leucovorin	13 (3.6)	0	92.3	7.7	0	0	0
Lung cancer							
Carboplatin + paclitaxel	117 (18.7)	0.9	0	89.7	9.4	0	0
Carboplatin + pemetrexed	114 (18.2)	0	0	100	0	0	0
Carboplatin + etoposide	100 (16.0)	0	0	66.0	34.0	0	0
Carboplatin + gemcitabine	45 (7.2)	0	0	95.6	2.2	2.2	0
Cisplatin + pemetrexed	37 (5.9)	0	0	100	0	0	0
NHL							
Cyclophosphamide + doxorubicin + rituximab + vincristine	39 (42.9)	0	0	97.4	2.6	0	0
Bendamustine + rituximab	15 (16.5)	0	0	13.3	86.7	0	0

NHL: non-Hodgkin lymphoma; QW: once weekly; Q2W: every 2 weeks; Q3W: every 3 weeks; Q4W: every 4 weeks.

[a]Only regimens administered to ≥11 patients included.

Table S3. FN Risk Classification for Planned Chemotherapy Regimens Among Patients With Metastatic Breast Cancer

	Periodicity				
**Planned Regimen**	**QW**	**Q2W**	**Q3W**	**Q4W**	**Unknown/Other**
Bevacizumab + Paclitaxel	Exclude	Low	Low	Low	Low
Carboplatin + Docetaxel	Exclude	As specified[a]	As specified[a]	As specified[a]	As specified[a]
Carboplatin + Docetaxel + Trastuzumab	Exclude	As specified[a]	High	High	per table
Carboplatin + Paclitaxel	Exclude	As specified[a]	Intermediate	As specified[a]	As specified[a]
Cyclophosphamide + Docetaxel	Exclude	High	High	High	High
Cyclophosphamide + Docetaxel + Doxorubicin	Exclude	High	High	High	High
Cyclophosphamide + Doxorubicin	Exclude	High	Intermediate	Intermediate	Intermediate
Cyclophosphamide + Doxorubicin + Paclitaxel	Exclude	High	Intermediate	Intermediate	Intermediate
Cyclophosphamide + Doxorubicin + Paclitaxel + /- Trastuzumab	Exclude	High	Intermediate	Intermediate	Intermediate
Paclitaxel	Exclude	High	Intermediate	Low	Intermediate
Paclitaxel + Trastuzumab	Exclude	High	High	High	High
Trastuzumab	Exclude	Exclude	Exclude	Exclude	Exclude
5-Fluorouracil + Cyclophosphamide	Unclassified	Unclassified	Unclassified	Unclassified	Unclassified
5-Fluorouracil + Cyclophosphamide + Docetaxel + Epirubicin	Unclassified	Unclassified	Unclassified	Unclassified	Unclassified
5-Fluorouracil + Cyclophosphamide + Doxorubicin	Unclassified	Unclassified	Unclassified	Unclassified	Unclassified
5-Fluorouracil + Cyclophosphamide + Epirubicin	Unclassified	Unclassified	Unclassified	Unclassified	Unclassified
Bevacizumab + Carboplatin + Paclitaxel	Unclassified	Unclassified	Unclassified	Unclassified	Unclassified
Bevacizumab + Carboplatin + Pemetrexed	Unclassified	Unclassified	Unclassified	Unclassified	Unclassified
Bevacizumab + Cyclophosphamide + Doxorubicin + Paclitaxel Starting Cycle 5	Unclassified	Unclassified	Unclassified	Unclassified	Unclassified
Bevacizumab + Docetaxel	Unclassified	Unclassified	Unclassified	Unclassified	Unclassified
Bevacizumab + Gemcitabine + Paclitaxel	Unclassified	Unclassified	Unclassified	Unclassified	Unclassified
Capecitabine	Unclassified	Unclassified	Unclassified	Unclassified	Unclassified
Capecitabine + Docetaxel	Unclassified	Unclassified	Unclassified	Unclassified	Unclassified
Capecitabine + Ixabepilone	Unclassified	Unclassified	Unclassified	Unclassified	Unclassified
Capecitabine + Ixabepilone + Zoledronic acid	Unclassified	Unclassified	Unclassified	Unclassified	Unclassified
Carboplatin + Cyclophosphamide + Doxorubicin + Paclitaxel	Unclassified	Unclassified	Unclassified	Unclassified	Unclassified
Carboplatin + Docetaxel + Pemetrexed + Trastuzumab	Unclassified	Unclassified	Unclassified	Unclassified	Unclassified
Carboplatin + Docetaxel + Pertuzumab + Trastuzumab	Unclassified	Unclassified	Unclassified	Unclassified	Unclassified
Carboplatin + Doxorubicin	Unclassified	Unclassified	Unclassified	Unclassified	Unclassified
Carboplatin + Gemcitabine	Unclassified	Unclassified	Unclassified	Unclassified	Unclassified
Carboplatin + Gemcitabine + Pamidronate	Unclassified	Unclassified	Unclassified	Unclassified	Unclassified
Cisplatin + Gemcitabine	Unclassified	Unclassified	Unclassified	Unclassified	Unclassified
Cisplatin + Pemetrexed	Unclassified	Unclassified	Unclassified	Unclassified	Unclassified
Cyclophosphamide + Docetaxel + Doxorubicin + Pemetrexed	Unclassified	Unclassified	Unclassified	Unclassified	Unclassified
Cyclophosphamide + Docetaxel + Doxorubicin + Pertuzumab	Unclassified	Unclassified	Unclassified	Unclassified	Unclassified
Cyclophosphamide + Docetaxel + Trastuzumab	Unclassified	Unclassified	Unclassified	Unclassified	Unclassified
Cyclophosphamide + Doxorubicin + Lapatinib + Paclitaxel Starting Cycle 5	Unclassified	Unclassified	Unclassified	Unclassified	Unclassified
Cyclophosphamide + Doxorubicin + Paclitaxel + Pemetrexed	Unclassified	Unclassified	Unclassified	Unclassified	Unclassified
Cyclophosphamide + Doxorubicin + Paclitaxel + Pertuzumab	Unclassified	Unclassified	Unclassified	Unclassified	Unclassified
Cyclophosphamide + Doxorubicin + Rituximab + Vincristine	Unclassified	Unclassified	Unclassified	Unclassified	Unclassified
Docetaxel	Unclassified	Unclassified	Unclassified	Unclassified	Unclassified
Docetaxel + Gemcitabine	Unclassified	Unclassified	Unclassified	Unclassified	Unclassified
Docetaxel + Pamidronate	Unclassified	Unclassified	Unclassified	Unclassified	Unclassified
Docetaxel + Pemetrexed + Trastuzumab	Unclassified	Unclassified	Unclassified	Unclassified	Unclassified
Docetaxel + Pertuzumab + Trastuzumab	Unclassified	Unclassified	Unclassified	Unclassified	Unclassified
Docetaxel + Trastuzumab	Unclassified	Unclassified	Unclassified	Unclassified	Unclassified
Doxorubicin + Gemcitabine + Vinorelbine	Unclassified	Unclassified	Unclassified	Unclassified	Unclassified
Eribulin	Unclassified	Unclassified	Unclassified	Unclassified	Unclassified
Fulvestrant + Gemcitabine + Paclitaxel + Palbociclib	Unclassified	Unclassified	Unclassified	Unclassified	Unclassified
Gemcitabine	Unclassified	Unclassified	Unclassified	Unclassified	Unclassified
Gemcitabine + Paclitaxel	Unclassified	Unclassified	Unclassified	Unclassified	Unclassified
Gemcitabine + Trastuzumab	Unclassified	Unclassified	Unclassified	Unclassified	Unclassified
Ixabepilone	Unclassified	Unclassified	Unclassified	Unclassified	Unclassified
Ixabepilone + Trastuzumab	Unclassified	Unclassified	Unclassified	Unclassified	Unclassified
Paclitaxel + Pamidronate	Unclassified	Unclassified	Unclassified	Unclassified	Unclassified
Paclitaxel + Pemetrexed	Unclassified	Unclassified	Unclassified	Unclassified	Unclassified
Paclitaxel + Pemetrexed + Trastuzumab	Unclassified	Unclassified	Unclassified	Unclassified	Unclassified
Palbociclib	Unclassified	Unclassified	Unclassified	Unclassified	Unclassified
Pertuzumab + Trastuzumab	Unclassified	Unclassified	Unclassified	Unclassified	Unclassified

Topotecan	Unclassified	Unclassified	Unclassified	Unclassified	Unclassified
Trastuzumab + Vinorelbine	Unclassified	Unclassified	Unclassified	Unclassified	Unclassified
Vinorelbine	Unclassified	Unclassified	Unclassified	Unclassified	Unclassified

FN: febrile neutropenia; high: projected risk >20%; intermediate: projected risk 10%–20%; low: projected risk <10%; QW: once weekly; Q2W: every 2 weeks; Q3W: every 3 weeks; Q4W: every 4 weeks.

[a]If the dose for either of the two drugs exceeds the threshold for high (carboplatin: AUC 6; docetaxel: 75 mg/m^2; paclitaxel: 175 mg/m^2), the regimen is classified as high. If the dose for either of the two drugs exceeds intermediate (but both are lower than high; carboplatin: AUC 5; docetaxel: 60 mg/m^2; paclitaxel: 135 mg/m^2), the regimen is classified as intermediate. Otherwise, the regimen is classified as low.

Table S4. FN Risk Classification for Planned Chemotherapy Regimens Among Patients With Metastatic Colorectal Cancer

	Periodicity				
**Planned Regimen**	**QW**	**Q2W**	**Q3W**	**Q4W**	**Unknown/Other**
5-Fluorouracil + 5-Fluorouracil (Cont) + Bevacizumab + Irinotecan + Leucovorin	Exclude	Low	Low	Low	Low
5-Fluorouracil + 5-Fluorouracil (Cont) + Bevacizumab + Leucovorin + Oxaliplatin	Exclude	Intermediate	Intermediate	Intermediate	Intermediate
5-Fluorouracil + 5-Fluorouracil (Cont) + Leucovorin + Oxaliplatin	Exclude	Intermediate	Intermediate	Intermediate	Intermediate
5-Fluorouracil + Leucovorin	Exclude	Low	Low	Low	Low
Bevacizumab + Capecitabine + Oxaliplatin	Exclude	Low	Low	Low	Low
Capecitabine	Exclude	Low	Low	Low	Low
Capecitabine + Oxaliplatin	Exclude	Low	Low	Low	Low
Oxaliplatin	Exclude	Low	Low	Low	Low
5-Fluorouracil (Cont)	Unclassified	Unclassified	Unclassified	Unclassified	Unclassified
5-Fluorouracil (Cont) + Bevacizumab + Oxaliplatin	Unclassified	Unclassified	Unclassified	Unclassified	Unclassified
5-Fluorouracil (Cont) + Capecitabine + Irinotecan + Leucovorin	Unclassified	Unclassified	Unclassified	Unclassified	Unclassified
5-Fluorouracil (Cont) + Cisplatin	Unclassified	Unclassified	Unclassified	Unclassified	Unclassified
5-Fluorouracil (Cont) + Irinotecan + Oxaliplatin	Unclassified	Unclassified	Unclassified	Unclassified	Unclassified
5-Fluorouracil (Cont) + Leucovorin + Oxaliplatin + Panitumumab	Unclassified	Unclassified	Unclassified	Unclassified	Unclassified
5-Fluorouracil (Cont) + Oxaliplatin	Unclassified	Unclassified	Unclassified	Unclassified	Unclassified
5-Fluorouracil + 5-Fluorouracil (Cont) + Bevacizumab + Capecitabine + Leucovorin + Oxaliplatin	Unclassified	Unclassified	Unclassified	Unclassified	Unclassified
5-Fluorouracil + 5-Fluorouracil (Cont) + Bevacizumab + Irinotecan + Leucovorin + Oxaliplatin	Unclassified	Unclassified	Unclassified	Unclassified	Unclassified
5-Fluorouracil + 5-Fluorouracil (Cont) + Capecitabine + Leucovorin + Oxaliplatin	Unclassified	Unclassified	Unclassified	Unclassified	Unclassified
5-Fluorouracil + 5-Fluorouracil (Cont) + Cetuximab + Irinotecan + Leucovorin	Unclassified	Unclassified	Unclassified	Unclassified	Unclassified
5-Fluorouracil + 5-Fluorouracil (Cont) + Cetuximab + Leucovorin + Oxaliplatin	Unclassified	Unclassified	Unclassified	Unclassified	Unclassified
5-Fluorouracil + 5-Fluorouracil (Cont) + Irinotecan + Leucovorin	Unclassified	Unclassified	Unclassified	Unclassified	Unclassified
5-Fluorouracil + 5-Fluorouracil (Cont) + Irinotecan + Leucovorin + Levoleucovorin + Oxaliplatin	Unclassified	Unclassified	Unclassified	Unclassified	Unclassified
5-Fluorouracil + 5-Fluorouracil (Cont) + Leucovorin	Unclassified	Unclassified	Unclassified	Unclassified	Unclassified
5-Fluorouracil + 5-Fluorouracil (Cont) + Leucovorin + Oxaliplatin + Panitumumab	Unclassified	Unclassified	Unclassified	Unclassified	Unclassified
5-Fluorouracil + 5-Fluorouracil (Cont) + Oxaliplatin	Unclassified	Unclassified	Unclassified	Unclassified	Unclassified
5-Fluorouracil + Bevacizumab + Leucovorin	Unclassified	Unclassified	Unclassified	Unclassified	Unclassified
5-Fluorouracil + Bevacizumab + Leucovorin + Oxaliplatin	Unclassified	Unclassified	Unclassified	Unclassified	Unclassified
5-Fluorouracil + Capecitabine + Irinotecan + Leucovorin + Panitumumab	Unclassified	Unclassified	Unclassified	Unclassified	Unclassified
5-Fluorouracil + Leucovorin + Oxaliplatin	Unclassified	Unclassified	Unclassified	Unclassified	Unclassified
Bevacizumab + Capecitabine	Unclassified	Unclassified	Unclassified	Unclassified	Unclassified
Bevacizumab + Capecitabine + Irinotecan	Unclassified	Unclassified	Unclassified	Unclassified	Unclassified
Bevacizumab + Irinotecan	Unclassified	Unclassified	Unclassified	Unclassified	Unclassified
Bevacizumab + Oxaliplatin	Unclassified	Unclassified	Unclassified	Unclassified	Unclassified
Capecitabine + Cetuximab + Irinotecan	Unclassified	Unclassified	Unclassified	Unclassified	Unclassified
Capecitabine + Cetuximab + Oxaliplatin	Unclassified	Unclassified	Unclassified	Unclassified	Unclassified
Capecitabine + Irinotecan + Oxaliplatin	Unclassified	Unclassified	Unclassified	Unclassified	Unclassified
Carboplatin + Etoposide	Unclassified	Unclassified	Unclassified	Unclassified	Unclassified
Carboplatin + Paclitaxel	Unclassified	Unclassified	Unclassified	Unclassified	Unclassified
Cetuximab	Unclassified	Unclassified	Unclassified	Unclassified	Unclassified
Cetuximab + Irinotecan	Unclassified	Unclassified	Unclassified	Unclassified	Unclassified
Cisplatin + Docetaxel + Etoposide	Unclassified	Unclassified	Unclassified	Unclassified	Unclassified
Cisplatin + Etoposide	Unclassified	Unclassified	Unclassified	Unclassified	Unclassified
Irinotecan	Unclassified	Unclassified	Unclassified	Unclassified	Unclassified

AUC area under the plasma drug concentration-time curve; FN: febrile neutropenia; high, projected risk >20%; intermediate, projected risk 10%–20%; low, projected risk <10%; QW: once weekly; Q2W: every 2 weeks; Q3W: every 3 weeks; Q4W: every 4 weeks.

Table S5. FN Risk Classification for Planned Chemotherapy Regimens Among Patients With Metastatic Lung Cancer

	Periodicity				
**Planned Regimen**	**QW**	**Q2W**	**Q3W**	**Q4W**	**Unknown/Other**
Bevacizumab + Carboplatin + Paclitaxel	Exclude	Intermediate	Intermediate	Intermediate	As specified[a]
Bevacizumab + Carboplatin + Pemetrexed	Exclude	Low	Low	Low	Low
Carboplatin + Docetaxel	Exclude	As specified[a]	High	Low	As specified[a]
Carboplatin + Etoposide	Exclude	Intermediate	Intermediate	Intermediate	Intermediate
Carboplatin + Gemcitabine	Exclude	Low	Low	Low	Low
Carboplatin + Paclitaxel	Exclude	As specified[a]	High	Low	As specified[a]
Carboplatin + Pemetrexed	Exclude	Low	Low	Low	Low
Cisplatin + Etoposide	Exclude	Intermediate	Intermediate	Intermediate	Intermediate
Cisplatin + Gemcitabine	Exclude	Low	Low	Low	Low
Cisplatin + Pemetrexed	Exclude	Low	Low	Low	Low
Cisplatin + Vinorelbine	Exclude	Exclude	Intermediate	Intermediate	Intermediate
Docetaxel	Unclassified	Unclassified	Unclassified	Unclassified	Unclassified
Pemetrexed	Exclude	Low	Low	Low	Low
Bendamustine + Rituximab	Unclassified	Unclassified	Unclassified	Unclassified	Unclassified
Bevacizumab + Carboplatin	Unclassified	Unclassified	Unclassified	Unclassified	Unclassified
Bevacizumab + Carboplatin + Paclitaxel + Pemetrexed	Unclassified	Unclassified	Unclassified	Unclassified	Unclassified
Bevacizumab + Carboplatin + Vinorelbine	Unclassified	Unclassified	Unclassified	Unclassified	Unclassified
Bevacizumab + Cisplatin + Gemcitabine	Unclassified	Unclassified	Unclassified	Unclassified	Unclassified
Bevacizumab + Cisplatin + Pemetrexed	Unclassified	Unclassified	Unclassified	Unclassified	Unclassified
Bevacizumab + Docetaxel + Gemcitabine	Unclassified	Unclassified	Unclassified	Unclassified	Unclassified
Bevacizumab + Gemcitabine	Unclassified	Unclassified	Unclassified	Unclassified	Unclassified
Bevacizumab + Gemcitabine + Oxaliplatin	Unclassified	Unclassified	Unclassified	Unclassified	Unclassified
Carboplatin	Unclassified	Unclassified	Unclassified	Unclassified	Unclassified
Carboplatin + Cetuximab + Gemcitabine	Unclassified	Unclassified	Unclassified	Unclassified	Unclassified
Carboplatin + Cetuximab + Paclitaxel	Unclassified	Unclassified	Unclassified	Unclassified	Unclassified
Carboplatin + Docetaxel + Gemcitabine	Unclassified	Unclassified	Unclassified	Unclassified	Unclassified
Carboplatin + Docetaxel + Paclitaxel	Unclassified	Unclassified	Unclassified	Unclassified	Unclassified
Carboplatin + Docetaxel + Pemetrexed	Unclassified	Unclassified	Unclassified	Unclassified	Unclassified
Carboplatin + Erlotinib + Paclitaxel	Unclassified	Unclassified	Unclassified	Unclassified	Unclassified
Carboplatin + Etoposide + Irinotecan	Unclassified	Unclassified	Unclassified	Unclassified	Unclassified
Carboplatin + Etoposide + Irinotecan + Paclitaxel + Topotecan	Unclassified	Unclassified	Unclassified	Unclassified	Unclassified
Carboplatin + Etoposide + Topotecan	Unclassified	Unclassified	Unclassified	Unclassified	Unclassified
Carboplatin + Gemcitabine + Paclitaxel	Unclassified	Unclassified	Unclassified	Unclassified	Unclassified
Carboplatin + Irinotecan	Unclassified	Unclassified	Unclassified	Unclassified	Unclassified
Carboplatin + Paclitaxel + Zoledronic acid	Unclassified	Unclassified	Unclassified	Unclassified	Unclassified
Cisplatin + Docetaxel	Unclassified	Unclassified	Unclassified	Unclassified	Unclassified
Cisplatin + Mannitol + Pemetrexed	Unclassified	Unclassified	Unclassified	Unclassified	Unclassified
Cyclophosphamide + Doxorubicin + Rituximab	Unclassified	Unclassified	Unclassified	Unclassified	Unclassified
Docetaxel + Gemcitabine	Unclassified	Unclassified	Unclassified	Unclassified	Unclassified
Docetaxel + Pemetrexed	Unclassified	Unclassified	Unclassified	Unclassified	Unclassified
Etoposide	Unclassified	Unclassified	Unclassified	Unclassified	Unclassified
Gemcitabine	Unclassified	Unclassified	Unclassified	Unclassified	Unclassified
Gemcitabine + Paclitaxel	Unclassified	Unclassified	Unclassified	Unclassified	Unclassified
Gemcitabine + Vinorelbine	Unclassified	Unclassified	Unclassified	Unclassified	Unclassified
Nivolumab	Unclassified	Unclassified	Unclassified	Unclassified	Unclassified
Paclitaxel	Unclassified	Unclassified	Unclassified	Unclassified	Unclassified
Topotecan	Unclassified	Unclassified	Unclassified	Unclassified	Unclassified
Vinorelbine	Unclassified	Unclassified	Unclassified	Unclassified	Unclassified

FN: febrile neutropenia; high: projected risk >20%; intermediate: projected risk 10%–20%; low: projected risk <10%; QW: once weekly; Q2W: every 2 weeks; Q3W: every 3 weeks; Q4W: every 4 weeks.

[a]If the dose for either of the two drugs exceeds the threshold for high (carboplatin: AUC 6; docetaxel: 75 mg/m^2; paclitaxel: 175 mg/m^2), the regimen is classified as high. If the dose for either of the two drugs exceeds intermediate (but both are lower than high; carboplatin: AUC 5; docetaxel: 60 mg/m^2; paclitaxel: 135 mg/m^2), the regimen is classified as intermediate. Otherwise, the regimen is classified as low.

Table S6. FN Risk Classification for Planned Chemotherapy Regimens Among Patients With NHL

Planned Regimen	Periodicity				
	QW	Q2W	Q3W	Q4W	Unknown/Other
Bendamustine + Rituximab	Exclude	Never	High	High	High
Cyclophosphamide + Doxorubicin + Rituximab + Vincristine	Exclude	High	Intermediate	Intermediate	Intermediate
Cyclophosphamide + Rituximab + Vincristine	Exclude	Intermediate	Intermediate	Intermediate	Intermediate
Alemtuzumab	Unclassified	Unclassified	Unclassified	Unclassified	Unclassified
Bendamustine	Unclassified	Unclassified	Unclassified	Unclassified	Unclassified
Bendamustine + Methylprednisolone + Rituximab	Unclassified	Unclassified	Unclassified	Unclassified	Unclassified
Bortezomib	Unclassified	Unclassified	Unclassified	Unclassified	Unclassified
Carboplatin + Etoposide + Ifosfamide + Rituximab	Unclassified	Unclassified	Unclassified	Unclassified	Unclassified
Chlorambucil	Unclassified	Unclassified	Unclassified	Unclassified	Unclassified
Cisplatin + Gemcitabine + Rituximab	Unclassified	Unclassified	Unclassified	Unclassified	Unclassified
Cyclophosphamide	Unclassified	Unclassified	Unclassified	Unclassified	Unclassified
Cyclophosphamide + Doxorubicin + Etoposide + Rituximab + Vincristine	Unclassified	Unclassified	Unclassified	Unclassified	Unclassified
Cyclophosphamide + Doxorubicin + Etoposide + Vincristine	Unclassified	Unclassified	Unclassified	Unclassified	Unclassified
Cyclophosphamide + Doxorubicin + Rituximab	Unclassified	Unclassified	Unclassified	Unclassified	Unclassified
Cyclophosphamide + Etoposide + Procarbazine + Rituximab	Unclassified	Unclassified	Unclassified	Unclassified	Unclassified
Cyclophosphamide + Etoposide + Rituximab	Unclassified	Unclassified	Unclassified	Unclassified	Unclassified
Cyclophosphamide + Etoposide + Rituximab + Vincristine	Unclassified	Unclassified	Unclassified	Unclassified	Unclassified
Cyclophosphamide + Fludarabine + Ofatumumab + Rituximab	Unclassified	Unclassified	Unclassified	Unclassified	Unclassified
Cyclophosphamide + Fludarabine + Rituximab	Unclassified	Unclassified	Unclassified	Unclassified	Unclassified
Etoposide + Ifosfamide + Mesna + Mitoxantrone + Rituximab	Unclassified	Unclassified	Unclassified	Unclassified	Unclassified
Fludarabine + Rituximab	Unclassified	Unclassified	Unclassified	Unclassified	Unclassified
Gemcitabine + Oxaliplatin + Rituximab	Unclassified	Unclassified	Unclassified	Unclassified	Unclassified
Gemcitabine + Rituximab	Unclassified	Unclassified	Unclassified	Unclassified	Unclassified
Rituximab + Temozolomide	Unclassified	Unclassified	Unclassified	Unclassified	Unclassified
Romidepsin	Unclassified	Unclassified	Unclassified	Unclassified	Unclassified

FN: febrile neutropenia; high: projected risk >20%; intermediate: projected risk 10%–20%; low: projected risk <10%; NHL: non-Hodgkin lymphoma; QW: once weekly; Q2W: every 2 weeks; Q3W: every 3 weeks; Q4W: every 4 weeks.

Table S7. Prophylaxis With G-CSF With Dosing Information During Chemotherapy Course Among Patients With Metastatic Cancer

	**All Cancers**			**Breast Cancer**			**Colorectal Cancer**			**Lung Cancer**			**NHL**		
	**High Risk (n=299)**	**IR + ≥1 Risk Factor (n=409)**	**All Others[a] (n=749)**	**High Risk (n=174)**	**IR + ≥1 Risk Factor (n=38)**	**All Others[a] (n=168)**	**High Risk (n=0)**	**IR + ≥1 Risk Factor (n=176)**	**All Others[a] (n=184)**	**High Risk (n=109)**	**IR + ≥1 Risk Factor (n=155)**	**All Others[a] (n=362)**	**High Risk (n=16)**	**IR + ≥1 Risk Factor (n=40)**	**All Others[a] (n=35)**
Any prophylaxis with G-CSF during course, %															
Primary prophylaxis	48.5	13.9	11.1	80.5	23.7	20.2	–	1.7	2.7	1.8	14.8	10.5	18.8	55	17.1
Secondary prophylaxis	9	12.2	9.2	10.9	23.7	7.7	–	11.9	6.5	5.5	9	9.7	12.5	15	25.7
Primary prophylaxis, %															
Pegfilgrastim	66.2	80.7	74.7	65	88.9	70.6	–	66.7	100	100	78.3	78.9	100	81.8	50
OBI	10.4	6.5	11.3	11	25	16.7	–	0	0	0	0	10	0	5.6	0
PFS/MDV	89.6	93.5	88.7	89	75	83.3	–	100	100	100	100	90	100	94.4	100
Filgrastim	33.8	19.3	25.3	35	11.1	29.4	–	33.3	0	0	21.7	21.1	0	18.2	50
Secondary prophylaxis, %															
Pegfilgrastim	63	52	56.5	57.9	44.4	46.2	–	42.9	58.3	66.7	78.6	60	100	33.3	55.6
OBI	11.8	7.7	7.7	0	0	0	–	11.1	0	25	9.1	9.5	50	0	20
PFS/MDV	88.2	92.3	92.3	100	100	100	–	88.9	100	75	90.9	90.5	50	100	80
Filgrastim	37	48	43.5	42.1	55.6	53.8	–	57.1	41.7	33.3	21.4	40	0	66.7	44.4
Primary prophylaxis, Pegfilgrastim															
OBI															
Total dose (mg), mean (SD)	6.0 (0)	6.0 (0)	6.0 (0)	6.0 (0)	6.0 (0)	6.0 (0)	–	–	–	–	–	6.0 (0)	–	6.0 (–)	–
Day of administration, %															
Day 1	0	0	0	0	0	0	–	–	–	–	–	0	–	0	–
Days 2–4	100	100	100	100	100	100	–	–	–	–	–	100	–	100	–
Days 5–6	0	0	0	0	0	0	–	–	–	–	–	0	–	0	–
PFS/MDV															
Total dose (mg), mean (SD)	6.0 (0)	6.0 (0)	6.0 (0)	6.0 (0)	6.0 (0)	6.0 (0)	–	6.0 (0)	6.0 (0)	6.0 (0)	6.0 (0)	6.0 (0)	6.0 (0)	6.0 (0)	6.0 (0)
Day of administration, %															
Day 1	1.2	2.3	5.5	1.2	0	5	–	0	0	0	5.6	7.4	0	0	0
Days 2–4	98.8	97.7	92.7	98.8	100	95	–	100	100	100	94.4	88.9	100	100	100
Days 5–6	0	0	1.8	0	0	0	–	0	0	0	0	3.7	0	0	0
Primary prophylaxis, Filgrastim															
Total dose (µg), mean (SD)	339.2 (74.0)	349.1 (84.1)	351.4 (100.9)	339.2 (74.0)	300 (–)	336.0 (113.8)	–	300 (–)	–	–	300.0 (0)	322.5 (63.6)	–	435.0 (90.0)	480.0 (0)
Number of days, mean (SD)	8.1 (2.0)	6.2 (3.1)	6.1 (3.1)	8.1 (2.0)	5.0 (–)	6.6 (2.8)	–	7.0 (–)	–	–	6.2 (3.6)	4.8 (3.2)	–	6.3 (3.8)	8.3 (2.9)
Secondary prophylaxis, Pegfilgrastim															
OBI															
Total dose (mg), mean (SD)	6.0 (0)	6.0 (0)	6.0 (0)	–	–	–	–	6.0 (–)	–	6.0 (–)	6.0 (–)	6.0 (0)	6.0 (–)	–	6.0 (–)

Day of administration, %															
Day 1	0	0	0	0	–	–	–	0	–	0	0	0	0	–	0
Days 2–4	100	100	100	100	–	–	–	100	–	100	100	100	100	–	100
Days 5–6	0	0	0	0	–	–	–	0	–	0	0	0	0	–	0
PFS/MDV															
Total dose (mg), mean (SD)	6.0 (0)	6.0 (0)	6.0 (0)	6.0 (0)	6.0 (0)	6.0 (0)	–	6.0 (0)	6.0 (0)	6.0 (0)	6.0 (0)	6.0 (0)	6.0 (–)	6.0 (0)	6.0 (0)
Day of administration, %															
Day 1	6.7	12.5	0	9.1	0	0	–	25	0	0	10	0	0	0	0
Days 2–4	93.3	87.5	100	90.9	100	100	–	75	100	100	90	100	100	100	100
Days 5–6	0	0	0	0	0	0	–	0	0	0	0	0	0	0	0
Secondary prophylaxis, Filgrastim															
Total dose (mcg), mean (SD)	327.0 (85.4)	327.5 (70.7)	345.5 (87.3)	333.8 (95.5)	300.0 (0)	325.7 (68.0)	–	345.0 (81.4)	312.0 (107.3)	300.0 (0)	300.0 (0)	369.2 (91.1)	–	330.0 (103.9)	345.0 (90.0)
Number of days, mean (SD)	5.9 (3.4)	5.2 (2.9)	4.1 (1.9)	6.4 (3.6)	3.8 (1.3)	4.1 (1.1)	–	5.0 (3.5)	4.4 (2.6)	4.0 (1.4)	5.0 (0)	4.2 (1.7)	–	7.5 (2.1)	3.5 (3.0)

G-CSF: granulocyte colony-stimulating factor; IR: intermediate risk; LR: low risk; MDV: multidose vial; NHL: non-Hodgkin lymphoma; OBI: on-body injector; PFS: prefilled syringe; UR: unknown risk.

[a]IR Chemotherapy + 0 Risk Factors or LR/UR Chemotherapy.

Table S8. FN incidence And Associated Outcomes Among Patients With Metastatic Cancer Not Receiving CSF Prophylaxis In Cycle 1

	All Cancers			Breast Cancer			Colorectal Cancer			Lung Cancer			NHL		
	High Risk	IR + ≥1 Risk Factor	All Others[a]	High Risk	IR + ≥1 Risk Factor	All Others[a]	High Risk	IR + ≥1 Risk Factor	All Others[a]	High Risk	IR + ≥1 Risk Factor	All Others[a]	High Risk	IR + ≥1 Risk Factor	All Others[a]
Course	n=154	n=352	n=666	N=34	n=29	n=134	n=0	n=173	n=179	n=107	n=132	n=324	n=13	n=18	n=29
Overall, %	16.9	15.9	14.3	20.6	10.3	14.9	–	15.0	12.3	16.8	18.2	14.2	7.7	16.7	24.1
Number of events, mean (SD)	1.1 (0.3)	1.1 (0.5)	1.2 (0.5)	1.1 (0.4)	1.0 (0)	1.3 (0.6)	–	1.1 (0.4)	1.1 (0.4)	1.1 (0.3)	1.3 (0.7)	1.1 (0.3)	1.0 (–)	1.0 (0)	1.9 (1.2)
Inpatient, %	96.2	89.3	95.8	85.7	100	95.0	–	80.8	90.9	100	95.8	97.8	100	100	100
Length of Stay, mean (SD)	6.7 (5.5)	5.1 (2.8)	6.5 (11.3)	4.6 (2.0)	5.0 (1.7)	6.7 (5.8)	–	4.7 (2.2)	6.8 (5.4)	7.3 (6.3)	5.0 (3.0)	5.7 (15.0)	9.0 (–)	8.7 (4.2)	9.3 (8.1)
Mortality, %	26.9	7.1	12.6	0	0	5.0	–	0	4.5	38.9	16.7	19.6	0	0	14.3
Outpatient, %	3.8	10.7	4.2	14.3	0	5.0	–	19.2	9.1	0	4.2	2.2	0	0	0
Cycle 1	n=154	n=352	n=666	n=34	n=29	n=134	n=0	n=173	n=179	n=107	n=132	n=324	n=13	n=18	n=29
Overall, %	7.8	4.8	5.3	8.8	0	7.5	–	1.7	3.4	7.5	9.1	5.6	7.7	11.1	3.4
Inpatient, %	100	94.1	97.1	100	–	90.0	–	66.7	100	100	100	100	100	100	100
Length of Stay, mean (SD)	6.8 (3.6)	4.8 (2.7)	7.6 (8.1)	6.0 (1.7)	–	6.9 (9.0)	–	4.5 (0.7)	9.0 (6.4)	6.9 (4.3)	4.3 (2.2)	7.5 (8.8)	9.0 (–)	8.0 (5.7)	7.0 (–)
Mortality, %	33.3	17.6	11.4	0	–	0	–	0	0	50.0	25.0	22.2	0	0	0
Outpatient, %	0	5.9	2.9	0	–	10.0	–	33.3	0	0	0	0	0	0	0
Cycle 2	n=138	n=329	n=591	n=34	n=28	n=123	n=0	n=171	n=161	n=92	n=112	n=281	n=12	n=18	n=26
Overall, %	3.6	4.0	3.2	2.9	3.6	3.3	–	4.1	2.5	4.3	4.5	2.8	0	0	11.5
Inpatient, %	100	76.9	100	100	100	100	–	57.1	100	100	100	100	–	–	100
Length of Stay, mean (SD)	7.0 (4.6)	5.0 (2.5)	6.5 (3.7)	4.0 (–)	4.0 (–)	8.3 (3.3)	–	6.0 (1.4)	5.3 (6.6)	7.8 (5.0)	4.4 (3.4)	6.3 (3.1)	–	–	6.3 (1.2)
Mortality, %	40.0	0	10.5	0	0	0	–	0	25.0	50.0	0	0	–	–	33.3
Outpatient, %	0	23.1	0	0	0	0	–	42.9	0	0	0	0	–	–	0
Cycle 3	n=115	n=312	n=523	n=33	n=26	n=107	n=0	n=167	n=149	n=70	n=102	n=243	n=12	n=17	n=24
Overall, %	4.3	2.9	2.1	6.1	3.8	2.8	–	3.6	0	4.3	2.0	2.5	0	0	8.3
Inpatient, %	100	77.8	90.9	100	100	100	–	66.7	–	100	100	83.3	–	–	100
Length of Stay, mean (SD)	7.0 (11.2)	3.7 (2.8)	6.7 (4.5)	2.5 (0.7)	7.0 (–)	6.0 (1.0)	–	4.0 (2.9)	–	10.0 (14.7)	1.5 (0.7)	7.8 (5.8)	–	–	5.0 (5.7)
Mortality, %	0	11.1	27.3	0	0	33.3	–	0	–	0	50.0	33.3	–	–	0
Outpatient, %	0	22.2	9.1	0	0	0	–	33.3	–	0	0	16.7	–	–	0
Cycle ≥4	n=102	n=292	n=462	n=31	n=22	n=94	n=0	n=162	n=137	n=60	n=94	n=209	n=11	n=14	n=22
Overall, %	6.9	7.5	9.5	6.5	4.5	8.5	–	7.4	9.5	8.3	8.5	8.6	0	7.1	22.7
Inpatient, %	85.7	90.9	95.5	50.0	100	100	–	91.7	84.6	100	87.5	100	–	100	100
Length of Stay, mean (SD)	4.7 (1.0)	6.1 (4.8)	5.6 (15.3)	3.0 (–)	5.5 (2.1)	8.4 (7.8)	–	4.5 (2.3)	5.6 (4.4)	5.0 (0.7)	8.4 (7.3)	3.1 (22.1)	–	10.0 (–)	10.3 (9.7)
Mortality, %	14.3	0	6.8	0	0	0	–	0	0	20.0	0	16.7	–	0	0
Outpatient, %	14.3	9.1	4.5	50.0	0	0	–	8.3	15.4	0	12.5	0	–	0	0

CSF: colony-stimulating factor; FN: febrile neutropenia; IR: intermediate risk; LR: low risk; NHL: non-Hodgkin lymphoma.

[a]IR Chemotherapy + 0 Risk Factors or LR/UR Chemotherapy.

Fig. S1. Schematic of chemotherapy course example in which a patient received only two cycles, all chemotherapy was administered on cycle day 1, both cycles included pegfilgrastim prophylaxis on cycle day 2, and cycle periodicity

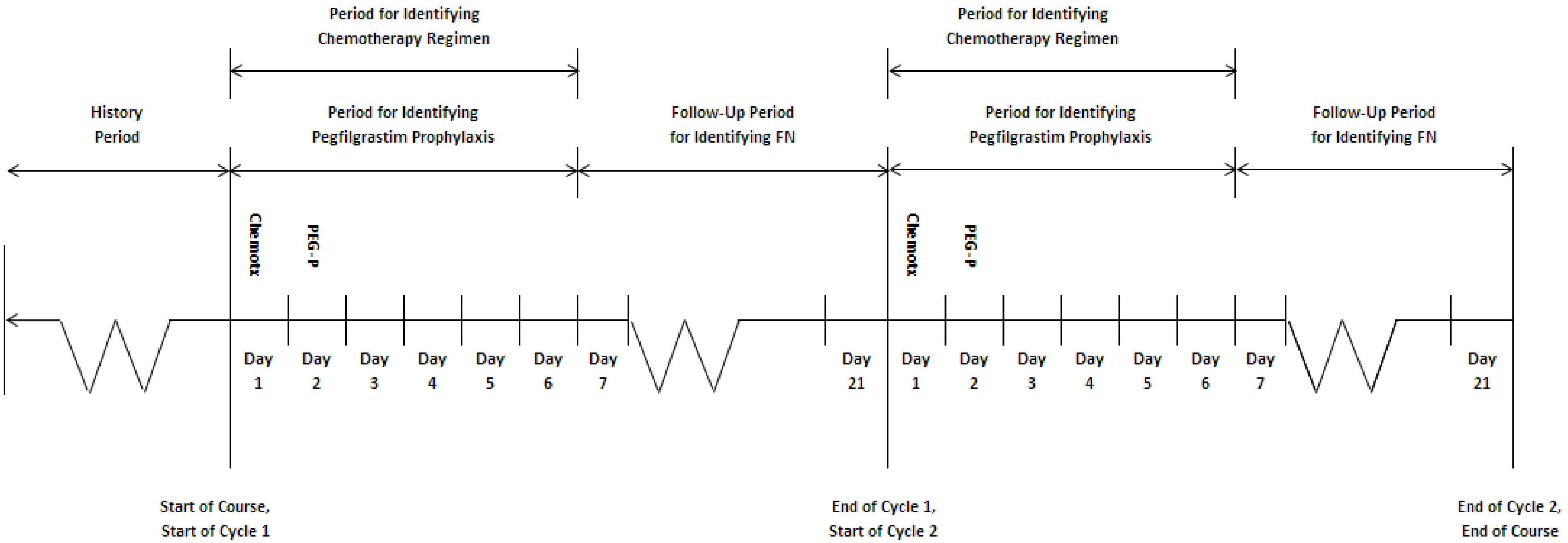

Chemotx, chemotherapy treatment; FN, febrile neutropenia; PEG-P, pegfilgrastim prophylaxis
